# Metabolomics-based biomarkers of fermented dairy and red meat intake: a randomized controlled trial in healthy adults

**DOI:** 10.3389/fchem.2024.1461331

**Published:** 2024-09-24

**Authors:** Giorgia La Barbera, Giulia Praticò, Lars Ove Dragsted, Catalina Cuparencu

**Affiliations:** Department of Nutrition, Exercise and Sports, University of Copenhagen, Copenhagen, Denmark

**Keywords:** metabolomics, biomarkers of food intake, dairy, red meat, microbial metabolites, liquid chromatography, high-resolution mass spectrometry

## Abstract

**Background:**

Dietary assessment is usually performed through imprecise tools, leading to error-prone associations between diet and health-related outcomes. Metabolomics has been applied in recent years to develop biomarkers of food intake (BFIs) and to study metabolites in the diet-microbiome crosstalk. Candidate BFIs exist to detect intake of meat and to a lesser extent dairy, but validation and further development of BFIs are needed. Here, we aim to identify biomarkers that differentiate between intakes of red meat and dairy, to validate previously reported BFIs for these foods, and to explore the effect of protein-matched meals on selected microbial metabolites.

**Methods:**

We conducted a randomized, controlled, cross-over single-meal study comparing a meal with highly fermented yogurt and cheese, and a meal with beef and pork meatballs. Postprandial urine samples from 17 subjects were collected sequentially after each meal up to 24 h and analyzed by untargeted metabolomics through ultra-high-performance-liquid chromatography (UHPLC) coupled via electrospray (ESI) source to a qTOF mass spectrometer. Univariate (repeated measures ANOVA) and multivariate (PLSDA, ML-PLSDA) data analyses were used to select BFIs differentiating the two meals. 3-Indoxyl sulfate, p-cresol sulfate, and several other microbial amino acid catabolites were additionally explored within the urine profiles.

**Results:**

Thirty-eight markers of meat and dairy intake were selected and are presented along with their excretion kinetics. Carnosine, taurine, and creatine, as well as hydroxyproline-based dipeptides are confirmed as meat BFIs. For dairy, previously reported metabolites such as acyl-glycines are confirmed, while proline-based dipeptides are reported as novel putative BFIs. Microbial metabolites showed only marginal evidence of differential formation after the two meals.

**Conclusion:**

This study allowed us to validate the postprandial kinetics of previously suggested biomarkers of meat and dairy intake and to identify new potential biomarkers. The excretion kinetics are useful to ensure that the collection of urine covers the correct time window in future dietary studies. The BFIs add to the existing body of biomarkers and may further be used in combination to provide a more reliable assessment of meat and dairy intake. Proteolytic microbial metabolites should be further investigated to assess the effect of different protein sources on health.

## 1 Introduction

Meat and dairy are important sources of high-quality protein. Yet, planetary health considerations point to the need for a reduction in the consumption of red meat and other sources of animal protein, leading to major dietary transitions in many regions across the world over the coming decades. During the green protein transition, monitoring the intake of different protein sources is important to understand its health effects. Dietary assessment is currently performed through 24-h recalls, food frequency questionnaires, and weighed food diaries (Bingham et al., 1994; Kipnis et al., 2002), that are known to provide only moderately reliable information (Neuhouser et al., 2008). More objective tools for dietary assessment are needed to prove associations between diet and health-related outcomes.

While red and processed meat consumption has been associated with an increased risk of colorectal cancer (Bouvard et al., 2015) and, possibly, cardiovascular diseases (Abete et al., 2014) and type 2 diabetes (Gu et al., 2023), a recent study using a biomarker-calibrated dietary assessment found no such associations (Zheng et al., 2022). Dairy is more generally associated with neutral or somewhat beneficial effects on health, especially for cardiometabolic outcomes (Dehghan et al., 2018), but the lack of good biomarkers of intake of dairy precludes a more accurate estimation of the magnitude of health benefits or adverse outcomes. Substituting animal with plant-based protein is generally associated with improved health (Neuenschwander et al., 2023), but considering the lower digestibility of plant proteins compared to animal protein (Ajomiwe et al., 2024) this seems like a paradox. Biomarker-based studies are therefore highly needed to gain novel insights regarding protein sources and their shorter and longer-term effects on health.

Metabolomics profiling of human samples by ultra-high performance liquid chromatography coupled to high-resolution mass spectrometry (UHPLC-HRMS), has enabled the outburst of new tools in dietary assessment, namely, biomarkers of food intake (BFIs) (Cuparencu et al., 2024). Several metabolites have been previously identified after intake of dairy products (Münger et al., 2018), including metabolites belonging to phenylalanine and tyrosine catabolic pathways, several aliphatic acid glycine conjugates, aromatic lactic acids, and some galactose derivatives. A combined BFI panel has been proposed and validated in free-living individuals (Li et al., 2021). However, its reproducibility across different labs has not been assessed and the thorough validation of other dairy markers is required. In contrast, biomarkers of meat intake have been extensively studied, and while well-validated biomarkers exist for overall meat as well as chicken intake, no specific red meat marker has been reported to date. BFIs specifically differentiating meat and dairy intakes, which are the major sources of animal protein, are also lacking. BFI validity covers biological and analytical aspects (Dragsted et al., 2018) and depends on several study-specific factors such as the dietary comparator, food matrix, variability of the food composition, the individual variability of metabolism and kinetics, and the timing of food intake and sample collection (Cuparencu et al., 2024). In most biomarker studies the diet is highly controlled to improve chances of finding new markers. In turn, this challenges the implementation of BFIs as part of complex unrestricted diets.

Differentiating between protein sources is also interesting in relation to microbial metabolism. While amino acids like tryptophan, phenylalanine, and tyrosine are readily available for the host, they are also readily available for the gut microbiota (Dodd et al., 2017). As a result of proteolytic fermentation, microbial metabolites are formed in the gut, including p-cresol conjugates and indole derivatives that were shown to contribute to both health and disease (Roager and Dragsted, 2019). For example, indole produced in the gut is a uremic toxin that is known to be elevated in kidney disease (Niwa, 2010; Schroeder et al., 2010). On the other hand, tryptophan can be metabolized by certain microbes into indolelactic acid, indolepropionic acid, or indoleacetic acid, with documented effects on barrier function and intestinal permeability, which in turn affect Ah2 receptors to downgrade inflammation (Roager and Dragsted, 2019) and consequently reduce cardiometabolic risk (De Mello et al., 2017; Xue et al., 2022; Zhang et al., 2022). While other food components, namely, dietary fibers, are known to further influence the microbiota activity and favor the formation of indolepropionic acid (Sinha et al., 2024), little is known about the contribution of meat and/or dairy intake to the short-term gut production of these metabolites.

In this work, we aim to identify BFIs for meat and dairy in a single meal cross-over study that represents more natural conditions, i.e., volunteers were not asked to restrict intakes of these foods preceding the test day, except for an overnight fast. A further aim was to validate the robustness of BFIs observed previously for meat or dairy intake, and to explore potential differences between the two protein sources in microbial protein catabolites during the 24 h after animal protein rich meals.

## 2 Materials and methods

### 2.1 Reagents and materials

Acetonitrile, methanol, and isopropanol (Optima grade) were purchased from Fisher Scientific (Pittsburgh, PA, United States). Formic acid (CAS 64-18-6), carnosine (CAS 305-84-0), taurine (CAS 107-35-7), creatine (CAS 6020-87-7), N6,N6,N6-Trimethyl-L-lysine (CAS 55528-53-5), 4-hydroxyphenylacetic acid (CAS:156-38-7), phenylacetyl-glycine (CAS 500-98-1), phenyllactic acid (CAS 828-01-3), isobutyryl-glycine (CAS 15926-18-8), isovaleryl-glycine (CAS 16284-60-9), p-cresol sulfate (CAS 3233-58-7), p-cresol glucuronide (CAS 17680-99-8), 3-indoxyl sulfate (CAS 2642-37-7), 3-indoxyl glucuronide (CAS 35804-66-1), indolelactic acid (CAS 832-97-3), phenylacetyl-glutamine (CAS 28047-15-6), tyramine (CAS 51-67-2), 4-ethylphenol (CAS 123-07-9), 3-ethylphenol (CAS 620-17-7), cyclohexane carboxylic acid (CAS 98-89-5), phenol (CAS 108-95-2), p-cresol (CAS: 106-44-5), propyl paraben (CAS 94-13-3), TRIS, NaOH, adenosine 3′-phosphate 5′-phosphosulfate lithium salt hydrate (PAPS) (CAS 109434-21-1), acetic anhydride (CAS 108-24-7), L-glutamine (CAS 56-85-9), and pooled human liver S9 extract were ordered from Merck (Darmstadt, Germany). Tiglyl-glycine (CAS 35842-45-6) was ordered from Santa Cruz Biotechnology (Heidelberg, Germany). 96-Well collection plates for collection and subsequent sample analysis were bought from Waters Corporation (Hedenhusene, Denmark). The chromatographic column HSS T3 (C18, 100 × 2.1 mm^2^, 1.8 μm particle size) and the VanGuard HSS T3 C18 column (2.1 × 5 mm^2^, 1.8 μm) were purchased from Waters Corporation (Hedenhusene, Denmark). Reagent water was ion-exchanged and purified further by a Millipore (Billerica, MA, United States) unit to obtain an electrical resistance below 18 MΩcm.

### 2.2 Synthesis of reference standards

Cyclohexane carboxylic acid glycine and 4-Hydroxyphenylacetyl-glutamine were synthesized according to the following protocol: 6 µmol of the substrate were mixed with 1 µmol of acetic anhydride and the solution was stirred at 35°C for 1 h. Glycine or glutamine (1 µmol) was added to the solution. After mixing, 2 µmol of NaOH were added to the solution and the solution was stirred at 35°C for 3 h. At the end of the reaction, NaOH was added drop by drop to get a final pH around 7. A negative control was also carried out by substituting the substrate with water.

A biomimetic enzymatic synthesis was used for the conjugation of sulfate to p-cresol, tyramine, 4-ethylphenol, 3-ethylphenol, phenol, and propylparaben. One mmol of the substrate and 23 µL of PAPS (C = 1 mgmL^−1^) were added to TRIS C = 500 μM at pH 7.5 and stirred at 37°C for 5 min. Twelve µL of chilled human liver S9 extract (C = 2 mg/mL) was thereafter added and the mixture was stirred again at 37°C for 1 h. Ice-cold MeOH was added in a 2:1 (*v/v*) excess to terminate the reaction. The mixture was centrifuged at 10,000 g for 4 min at 5°C. The supernatant was collected and evaporated to dryness. The dried sample was redissolved in 200 μL H_2_O:ACN = 90:10 (*v/v*). Formation of p-cresol sulfate from p-cresol was used as a positive control to confirm the activity of the enzyme system. A negative control was also carried out by substituting the substrate by 5 µL of C = 500 µM TRIS (pH 7.5).

Pyroglutamyl-proline was synthesized according to the protocol provided by Stanstrup et al. (2014a).

### 2.3 Study design

The study was approved by the Danish National Committee on Biomedical Research Ethics of the Capital Region of Denmark (J. No. H-3-2012-151), registered in the US National Library of Medicine (NCT01773304), and carried out at the Department of Nutrition, Exercise and Sports, Faculty of Science, University of Copenhagen, Frederiksberg (Denmark). Healthy men and women aged 18–50 years and with a body mass index (BMI) between 25 and 40 kg/m^2^ (overweight) were recruited from the Capital Region, Denmark. Participants were not included if, among others, they performed physical activity >10 h per week, were smoking, had a parental history of osteoporosis, used dietary supplements or medications, had chronic diseases, or were pregnant or breastfeeding. From the 24 initially recruited a total of 17 subjects (65% women) completed the study.

**FIGURE 1:**
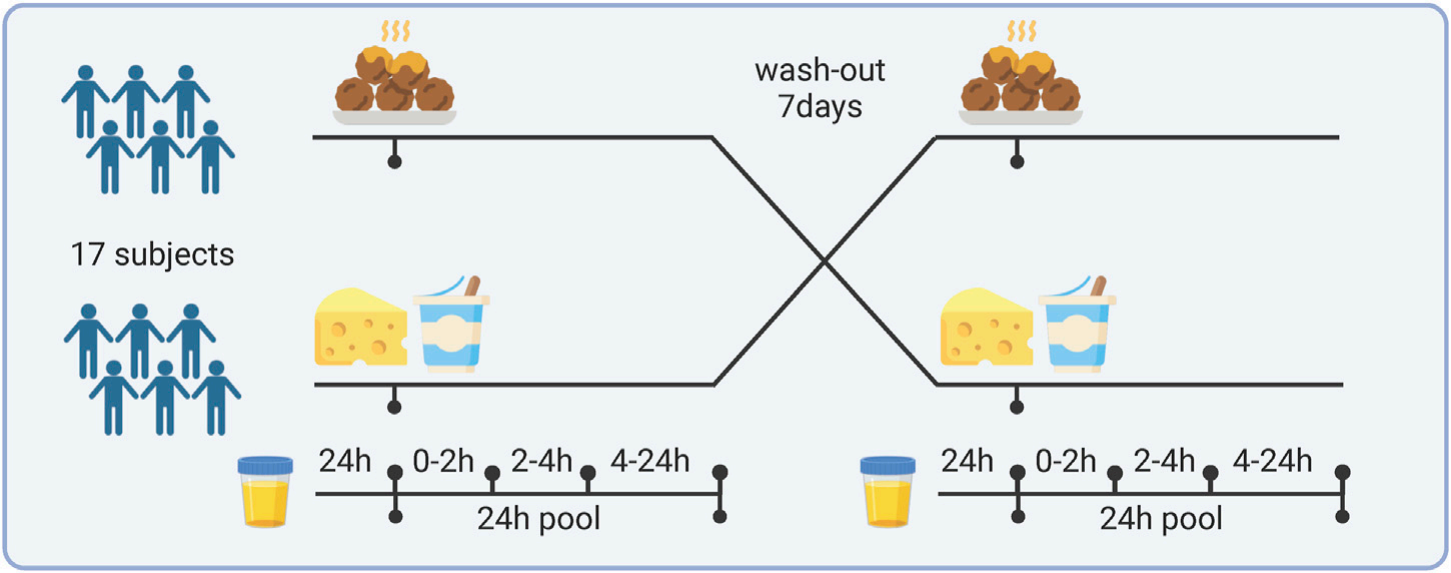
Study design and sample collection. Created with BioRender.com.

The study was designed as a randomized, cross-over intervention with 2 study periods of 48 h each and a wash-out period of 7 days between intervention periods as shown in Figure 1. Each period started with a 24-h urine collection representing the habitual diet of the day preceding the test day. On the test day, the subjects came in after an overnight fast (8–12 h) and were randomized to either a breakfast high in dairy or red meat protein. The dairy meal consisted of 250 g of a typical fruit-flavored skyr, which is a highly fermented yogurt product (flavor of raspberry, rhubarb, and vanilla), 170 g fermented cheese, 23 g white bread, and 330 g water. The red meat protein meal consisted of 340 g of minced meat balls made from 180 g beef and 160 g pork, and 330 g of a fruit drink to balance the fruit-flavored dairy meal (55 g blackcurrant juice (Ribena); 275 g water). Both test meals were matched in energy and contributed equal amounts of protein, fat, and carbohydrate. Six grams of Capolac (Arla Food Ingredients, Denmark) were added to the meat so that the calcium content reflected the content in the cheese meal (−1500 mg). Thus, the test meals differed only in type of protein and a minor difference in supplemented berry flavors. Twenty-four hours before the intervention visits, subjects were asked to restrain from hard physical activity, alcohol, fish, and fish products (to isolate potential effects on bone mass measurements, which was originally planned as a study outcome but never addressed), and to ingest the same (self-selected) dinner meals the night before each of the two intervention visits. After ingestion of each test meal, the subjects were asked to refrain from consuming any proteins of animal origin up until the next morning. Four urine samples were collected covering a total of 24 h, as follows: 1) from 24 h prior the intervention visit until 20 min before having the test meal (T 0), 2) from 0 until 2 h following intake of the test meal (T 0–2), 3) from 2 until 4 h after the test meal (T 2–4), and (4) during the rest of the day, including the first void of the next morning at around 7 a.m. (T 4–24). The subjects were instructed to keep the samples below 5°C in cooler bags or in a refrigerator during the collection days. Aliquots were collected from each sample and stored at −80^o^C. In addition, a pool of the total collections (T0-2, T2-4, and T4-24) was created and stored as well.

### 2.4 Urine sample preparation for LC-MS untargeted metabolomics

Urine samples were centrifuged (4°C, 4 min, 2,700 RCF) and 150 μL of each urine sample was added to a well in 96-well collection plates together with 150 μL of solvent (water with 5% 30:70 (v/v) ACN: MeOH) containing a solution with 7 Internal Standards as described previously (Barri et al., 2012). In addition, an external metabolite standard mixture with 44 different compounds was used for quality control of the analytical platform (Barri et al., 2012). A pooled sample containing equal amounts of all urine samples analyzed was prepared and added to separate wells on all plates for subsequent control of instrumental drift and batch effect in the data preprocessing and analysis. Samples from the same person were randomized within one plate to minimize intra-individual variation due to plate differences. After preparation the plates were sealed and kept at 4 °C if analyzed within the next 24 h; they were otherwise kept at −80°C and thawed and gently agitated prior to analysis.

### 2.5 UHPLC-HRMS analysis

The urine samples were analyzed on an ultra-high performance liquid chromatographic system (Acquity UHPLC, Waters, Manchester, United Kingdom) coupled through an electrospray interface (ESI) to orthogonal acceleration quadruple time-of-flight (Premier qTOF) mass-spectrometer (Waters, Manchester, United Kingdom). A sample volume of 5 μL was injected into a HSS T3 C18 column (ACQUITY HSS T3 C18 column, 2.1 × 100 mm, 1.8 µm, Milford, United States) with a VanGuard HSS T3 pre-column (ACQUITY VanGuard HSS T3 C18 column, 2.1 × 5 mm, 1.8 µm, Milford, United States), as described previously (Barri T et al., 2012). The mobile phase A consisted of H_2_O:HCOOH = 99.9:0.1 (*v/v*), the mobile phase B consisted of ACN:MeOH:HCOOH = 70:29.9:0.1 (*v/v/v*). A gradient of both mobile phase solvent and flow rate was used (run time 7 min). The linear concentration gradient was set as follows: start condition (5% B), 1 min (8% B), 2 min (15% B), 3 min (40% B), 4 min (70 %B), 4.5 min (100% B), 6.4 min (100% B), 6.6 min (5% B), 7 min (5% B). The linear flow gradient was set as follows: start condition (0.5 mLmin^−1^), 1 min (0.5 mLmin^−1^), 2 min (0.6 mLmin^−1^), 3 min (0.7 mLmin^−1^), 4 min (0.8 mLmin^−1^), 4.5 min (1 mLmin^−1^), 5 min (1.2 mLmin^−1^), 6.4 min 1.2 mLmin^−1^), 6.6 min (1 mLmin^−1^), 6.8 min (0.5 mLmin^−1^), 7 min (0.5 mLmin^−1^). ESI was used in both positive and negative acquisition modes during separate runs, with capillary probe voltages of 3.2 kV and 2.8 kV, respectively. For both modes the following parameters were set: ion source temperature 120°C, desolvation gas (nitrogen) temperature 400°C, cone voltage 25 kV, and cone and desolvation gas flows 50 and 1,000 L/h, respectively. Leucine enkephalin was infused every 10 s for 0.1 s as a lock-mass solution for continuous mass calibration. For both positive and negative polarity modes the selected mass range was from 50 to 1,000 *m/z* in full scan mode with a scan time of 0.08 s and an inter-scan delay of 0.02 s. Blanks (water with 5% ACN:MeOH 70:30 *v/v*) and external metabolomics standard mixtures were injected after every 30 samples, throughout each analytical batch. Pooled samples were injected after every 50 samples.

### 2.6 Data pre-processing

Raw data were converted to NetCDF using the DataBridge program converter (MassLynx V4.1, Waters Corporation, Manchester, United Kingdom). NetCDF files were imported into MZmine 2.16 (Pluskal et al., 2010) and preprocessed separately for positive and negative ionization modes including the following steps: mass detection (noise level: 60), chromatogram builder (RT tolerance: 0.01, *m/z* tolerance: 0.03, noise: 80), chromatogram deconvolution (local minimum search, ratio 1.3), isotopic peaks grouper, peak alignment (join aligner, RT tolerance: 0.05, *m/z* tolerance: 0.03), duplicate peak filter, peak list row filter, and gap filling (peak finder, RT tolerance 0.01, *m/z* tolerance 0.03). As a result, a matrix where the samples were displayed as rows and the height of aligned peaks as columns was obtained. Each detected peak was defined as a feature, consisting of a specific retention time and a mass to charge ratio (*m/z*). Processed data were imported into Matlab^®^ (MATLAB R2016a; MathWorks Inc., Natick, MA, 2000) and the features obtained were further filtered, as follows. Features were removed if: 1) they were present in blanks above noise level (>60 of intensity, or S/N > 6), 2) they eluted early (before 0.3 min) or late (after 6min), or 3) they had a peak height lower than 10 (considered as noise level or gap filling errors) in more than 60% of the samples within every sample group, for each time point (Bijlsma et al., 2006). Intensities were subsequently corrected feature-wise, so that the mean intensity of each sample was the same for each batch, thereby removing inter-batch variation, ensured by the previous randomization of each subject within the same batch. After a separate pre-processing of the data for the two ionization modes, the two resulting matrices were concatenated and features within a retention time range of 0.02 were grouped and assigned as one unique feature group if they had Pearson correlation coefficients higher than 0.7 to ease the identification process. Following this step, each feature group was considered as originating from the same compound. Prior to statistical data analysis, zero values were replaced by half the minimum recorded value for each feature, as previously described (Castillo et al., 2011). Data were normalized (PQN) and autoscaled before the analysis (Li et al., 2016).

### 2.7 Data analysis

Univariate analysis was performed using Matlab^®^ (MATLAB R2016a; MathWorks Inc., Natick, MA, 2000) on the urine samples collected before the test meal (T 0), during the first 2 (T 0-2), 4 (T 2-4) and 24 h (T4-24) after the test meal in order to identify the features discriminating the two meals, with a proper excretion profile over the 24 h following the test meal, defined as a monotonic trajectory to its peak. To evaluate the effect of the two meals over time on the urine metabolome, repeated measures ANOVA was employed where the effect of time and meal and their interaction were modeled as fixed factors, and subject-specific effects were included in the model as random factors. Features with a *p*-value<0.05 for the effect of meal and the interaction meal*time, that is features showing a significant difference between the meals as an effect modified by time, were kept for further analysis. For such features, adjusted *p*-values for multiple comparisons were calculated with the Dunn and Sidák’s approach (Šidák, 1967) to evaluate at which time point each feature showed a significant difference between the meals. Only the features showing a significant *p*-value < 0.05 for the comparison between the meals at least for two consecutive time points were considered for further biomarker investigation, e.g., T0-2 and T2-4 or T2-4 and T4-24.

Multivariate data analysis was performed on the pooled 24-h urine sample collected postprandially after each of the meals. Two different approaches were used to identify discriminating markers. At first, data were analyzed by Partial Least Square-Discriminant Analysis (PLSDA), carried out by PLS_Toolbox (version 6.5, eigenvector Research, Inc., MA, United States). To evaluate the discriminant features between the 2 meals in 24 h urine samples, PLSDA model has been computed on the combined matrix containing the features from both positive and negative ion mode, for a total of 5119 features by 34 samples (two pooled 24 h pools from 17 subjects). The PLSDA models have been cross-validated by employing 10 pairs of training sets for calibration (n = 30 samples) and test-sets for validation (n = 4) that were defined by randomly removing two subjects at a time, so that each set included data from both meals in a balanced way. Variable selection has been carried out on the 10 calibration sets by removing variables with selectivity ratio and variable importance in projection (VIP) values lower than 1 until no further increase in the cross-validation (8-fold) classification errors could be observed (Gürdeniz et al., 2016). The models computed with selected variables were evaluated using test set misclassification (Szymańska et al., 2012). The variables that were present at least in 80% of the calculated models were kept for further investigation.

As a second step, the same data were investigated by Multilevel Partial Least-Squares Discriminant Analysis (ML-PLSDA) to correct for the random effect of the subjects (Westerhuis et al., 2010). This approach uses the structure of the cross-over design to separate the contribution of the variance between and within the subjects. Using the routine available online at http://www.bdagroup.nl for ML-PLSDA, within-subject variation was extracted from autoscaled data, and a PLSDA model was carried out on the extracted matrix and validated as described above. Only features that significantly discriminated between the meals in the univariate data analysis and at least one of the two multivariate approaches were selected for identification.

Previously reported biomarkers for intake of dairy and meat, as well as proteolytic microbial metabolites were retrieved from the pre-processed data in the untargeted run. For dairy, these comprised of galactose, lactose, galactitol, galactonate (Münger et al., 2017) and the acyl-glycines isobutyryl-glycine, isovaleryl-glutamic acid, isovaleryl-glycine, and tiglyl-glycine (Hjerpsted et al., 2014). For meat these included anserine and 3-methylhistidine (Cuparencu et al., 2019a). Phenol sulfate, p-cresol sulfate and glucuronide, 3-indoxyl sulfate and glucuronide, phenylacetic acid, phenylacetyl-glutamine, tyramine, indolepropionic acid, indolelactic acid, and indoleacetic acid were targeted as microbial metabolites. These were investigated regardless of whether they were significantly different by any statistical approach.

Lastly, correlations between newly discovered as well as targeted BFIs and microbial metabolites were performed by Pearson correlation and illustrated in a correlation map (MATLAB R2021b; MathWorks Inc., Natick, MA, 2000). Correlation coefficients (r) between 0.40 and 0.69 were considered moderate, and >0.70 were considered strong (Schober and Schwarte, 2018).

### 2.8 Identification

The features resulting from the statistical analysis were investigated for the final identification of new BFIs of dairy and meat. Firstly, a search within an in-house database, containing retention time information and MS spectra of 400 reference substances, was carried out. Secondly, for unknown compounds with a signal area higher than 300, MS/MS spectra were recorded on the Premier qTOF to obtain structural information. The collision-induced dissociation (CID) was set to 10, 20, and 30 eV in separate runs, and all other parameters were kept as for the MS full scan experiments described above. To obtain spectra with higher mass accuracy for some of the markers, MS/MS fragmentation analyses were performed on a H Class UHPLC coupled to a Vion IMS qTOF mass spectrometer (Waters^®^). A reversed-phase column (ACQUITY HSS T3 C18 column, 2.1 × 100 mm, 1.8 µm, Milford, United States) coupled with a pre-column (ACQUITY VanGuard HSS T3 C18 column, 2.1 × 5 mm, 1.8 µm, Milford, United States) was used at a temperature of 50°C. The mobile phases consisted of 0.1% formic acid in water (solvent A), methanol (solvent B), 0.1% formic acid in 70:30 acetonitrile: methanol (solvent C), and isopropanol (solvent D). The duration of the analytical run was 10 min with the following flow rate: 0 min (0.4 mLmin^−1^), 0.75 min (0.4 mLmin^−1^), 6 min (0.5 mLmin^−1^), 6.5 min (0.5 mLmin^−1^), 8 min (0.6 mLmin^−1^), 8.1 min (0.4 mLmin^−1^), 9 min (0.4 mLmin^−1^), 10 min (0.4 mLmin^−1^), and the following gradient: 0 min (100% A), 0.75 min (100% A), 6 min (100% B), 6.5 min (70% C, 30% D), 8 min (70% C, 30% D), 8.1 min (70% C, 30% D), 9 min (100% A), 10 min (100% A). Targeted MS/MS was performed on selected urine samples at 10, 30, and 50 eV. Thereafter, parent ions and fragments were searched in different databases, such as Human Metabolome Database (http://www.hmdb.ca), Metlin (http://metlin.scripps.edu), Chemspider (http://www.chemspider.com), mzCloud (http://www.mzcloud.org), and *in silico* fragmentation software, i.e., SIRIUS (https://bio.informatik.uni-jena.de/software/sirius/) (Dührkop et al., 2019). As a last step, the identities of the metabolites were confirmed with authentic commercial standards. If unavailable, reference compounds were synthesized chemically or with biomimetic synthesis (see 2.2). The biomarkers were classified into four levels of identification confidence according to the criteria established by Sumner *et al.* as a standard for metabolomics reporting (Sumner et al., 2007). Briefly, level I was assigned to compounds matched to standards by means of two orthogonal criteria, i.e., RT and *m/z*; level II was assigned to compounds matched to an MS/MS spectrum published in the literature or spectral libraries; level III was assigned to compounds identified at the level of compound class by spectral similarity to known compounds of a specific chemical class and level IV was assigned to unknown compounds.

## 3 Results

### 3.1 Participant characteristics

We recruited 24 subjects for the study but 7 signed out due to lack of time or for personal reasons. The 17 participants who completed the study had a mean (range) age of 35.5 (19–49) years and a mean BMI (std. dev.) of 29.3 (3.6).

### 3.2 Data analysis

The data pre-treatment resulted in 2294 and 2825 features in positive and negative mode, respectively. By repeated measures-ANOVA evaluating the effect of the meal over time, 179 features showed a significant difference between meals for at least two consecutive time points. These were kept for further analysis. The two multivariate statistical approaches that employed the pooled 24-h collected after the meals, resulted in 52 features that discriminated between the two meals by PLSDA (Supplementary Table S1; Supplementary Figure S1) and 67 features discriminating between meals by ML-PLSDA (Supplementary Table S1; Supplementary Figure S2). The model characteristics are given in Supplementary Table S1. Seventy-eight features significantly discriminated the two meals by univariate analysis and one of the multivariate strategies; these were selected as the main features discriminating the two diets. The 78 features were further reduced to 38 metabolites after grouping of fragments and adducts.

In addition, 12 metabolites previously reported as dairy intake or microbial proteolytic biomarkers in the literature were found in the pre-processed data and added to the final list (Cuparencu et al., 2019b; Münger et al., 2018; Roager and Dragsted, 2019). The resulting 50 metabolites have been reported in Tables 1–3 along with their corresponding selection by data analyses strategies. A correlation map of the 50 metabolites is shown in Supplementary Figure S3 in the Supplementary Material.

**TABLE 1:** Markers of meat-based meal reported with their retention time (RT), putative identity and relative identification confidence level in apex, molecular formula, theoretical neutral mass, measured precursor ion mass, measured fragments and adduct ion masses, significance in the applied statistical methods, and excretion kinetics.

feature	RT	Metabolite (Level identification)	Molecular formula	Theoretical mass	Measured ion	Suggested ion	Fragments and adducts		ANOVA	ML PLSDA	PLSDA	Excretion kinetics
M01	0.46	carnosine^I^	C_9_H_14_N_4_O_3_	226.1066	225.0974	[M-H]^−^			*	*	*	A
M02	0.49	taurine^I^	C_2_H_7_NO_3_S	125.0146	124.0070	[M-H]^−^			*	*		B
M03	0.51	creatine^I^	C_4_H_9_N_3_O_2_	131.0649	132.0771	[M+H]^+^	170.0339	[M+K]^+^	*	*	*	B
					130.0604	[M-H]^−^	154.0603	[M+Na]^+^				
							263.1470	[2M+H]^+^				
							900.5499	[M+H-COCH_2_]^+^				
							880.3971	[M-H-COCH_2_]^−^				
M04	0.45	N6,N6,N6-Trimethyl-L-lysine^I^	C_9_H_20_N_2_O_2_	188.1525	189.1606	[M+H]^+^	211.1400	[M+Na]^+^	*	*	*	D
							130.0870	[M+H-C_3_H_9_N]^+^				
M05	0.54	Hydroxyprolyl-Proline^III^	C_10_H_16_N_2_O_4_	228.1110	227.1035	[M-H]^−^	209.1040	[M-H-H_2_O]^−^	*	*	*	B
							165.1020	[M-H-H_2_O-CO_2_]^−^				
							155.0810	[C_7_H_11_N_2_O_2_]^−^				
							130.0490	[HyP–H]^−^				
							127.0860	[C_6_H_11_N_2_O]^−^				
							68.0500	[C_4_H_6_N]^−^				
M06	0.54	Prolyl-Hydroxyproline^III^	C_10_H_16_N_2_O_4_	228.1110	229.1190	[M+H]^+^	132.0450	[HyP+H]^+^	*	*	*	C
							114.0460	[HyP-H_2_O+H]^+^				
							86.0440	[HyP-HCOOH+H]^+^				
							70.0540	[HyP-H_2_O-CO_2_+H]^+^				
							68.0400	[HyP-H_2_O-HCOOH+H]^+^				
M07	0.65	Leucyl/Isoleucyl-Hydroxyproline^III^	C_11_H_20_N_2_O_4_	244.1423	245.1493	[M+H]^+^	132.0790	[HyP+H]^+^	*	*	*	C
							114.0690	[HyP-H_2_O+H]^+^				
							86.0980	[Ile/Leu-HCOOH]^+^				
							86.0610	[HyP-HCOOH+H]^+^				
							68.0490	[HyP-HCOOH-H_2_O+H]^+^				
							58.0640	[C_3_H_8_N]^+^				
M08	1.26	Hydroxyprolyl-Leucine/Isoleucine^III^	C_11_H_20_N_2_O_4_	244.1423	245.1505	[M+H]^+^	227.1030	[M+H-H_2_O]^+^	*	*		B
							209.0550					
							191.0450					
							140.0700					
							114.0540	[HyP-H_2_O+H]^+^				
							100.0750	[C_5_H_10_NO]^+^				
							70.0630	[HyP-H_2_O-CO_2_+H]^+^				
							58.0640	[C_3_H_8_N]^+^				
M09	3.08	Prolyl-Phenylalanyl-Glycine^III^	C_16_H_21_N_3_O_4_	319.1532	320.1636	[M+H]^+^	245.1280	[M-Gly+H]^+^	*	*	*	B
							223.1070	[M-Pro-OH+H]^+^				
							217.1340	[M-Gly-CO+H]^+^				
							120.0800	[Phe-HCOOH+H]^+^				
							70.0640	[Pro-HCOOH+H]^+^				
M10	3.23	Acetyl-Leucine^II^	C_8_H_15_NO_3_	173.1052	172.0967	[M-H]^−^	130.0900	[Leu-H]^+^	*		*	C
M11	3.50	dihydroxybenzoic acid methyl ester sulfate^III^	C_8_H_8_O_7_S	247.9991	246.9944	[M-H]^−^	214.9640	[M-H-CH_3_OH]^−^	*	*	*	B
							186.9690	[M-H-CH_3_OH-CO]^−^				
							167.0340	[M-H-SO_3_]^−^				
							135.0080	[M-H-CH_3_OH-SO_3_]^−^				
							107.0130	[M-H-CH_3_OH-SO_3_-CO]^-^				
							79.0180	[M-H-CH_3_OH-SO_3_-2CO]^−^				
M12	3.66	Unknown sulfate^IV^	C_18_H_23_NO_7_S	397.1195	396.1104	[M-H]^−^	316.1549	[M-H-SO_3_]^−^	*	*	*	B
							228.0073					
							194.1174					
							16.8102	[C_9_H_14_NO_2_]^−^				
							150.0915	[C_9_H_12_NO]^−^				
							147.0447	[C_9_H_7_O_2_]^−^				
							140.0712	[C_7_H_10_NO_2_]^−^				
							112.0763	[C_6_H_10_NO]^−^				
							105.0338	[C_7_H_5_O]^−^				
							84.04521	[C_4_H_6_NO]^−^				
							79.95595	[SO_3_]^−^				
M13	3.69	Unknown^IV^	C_12_H_19_NO_3_	225.1365	226.1449	[M+H] ^+^	142.0850	[C_7_H_12_NO_2_]^+^	*	*	*	B
							135.0430	[C_8_H_7_O_2_]^+^				
							107.0490	[C_7_H_7_O]^+^				
							79.0530	[C_6_H_7_]^+^				
							77.0380	[C_6_H_5_]^+^				
							68.9970	[C_3_HO_2_]^+^				
M14	3.71	Unknown sulfate^IV^			398.1269	[M-H] ^−^	318.1700	[M-H-SO_3_]^−^	*	*	*	C
							300.1578	[M-H-H_2_O-SO_3_]^−^				
							79.9560	[SO_3_]^−^				
M15	3.32	2,3-Dihydro-3,5-dihydroxy-2-oxo-3-indoleacetic acid^III^	C_10_H_9_NO_5_	223.0481	222.0395	[M-H]^−^	178.0530	[M-CO_2_-H]^−^	*		*	B
							148.0470	[M-CO_2_-CH_2_O-H]^−^				
							121.0344	[M-CO_2_-CH_2_O-HCN-H]^-^				
							91.0228	[M-CO_2_-2CH_2_O-H]^−^				
M16	3.39	unknown ^IV^			259.0268	[M-H]^−^	179.0680	[M-SO_3_-H]^−^	*	*		A
							121.0280	[M-C_3_H_5_O-SO_3_-H]^−^				
							79.9570	[SO_3_]^−^				
							57.0346	[C_3_H_5_O]^−^				
M17	3.92	unknown^IV^	C_15_H_22_O	218.1671	219.1746	[M+H]^+^			*	*	*	C
M18	0.56	unknown^IV^	C_6_H_8_O_3_S	160.0194	159.0129	[M-H]^−^	11.5019	[M-H-CO_2_]^−^	*	*		B
							101.0040	[M-H-CO_2_CH_2_]^−^				
							78.9590	[SO_3_]^−^				
											

**TABLE 2:** Markers of dairy-based meal reported with their retention time (RT), putative identity and relative identification confidence level in apex, molecular formula, theoretical neutral mass, measured precursor ion mass, measured fragments and adduct ion masses, significance in the applied statistical methods, and excretion kinetics.

Feature	RT	Metabolite (level identification)	Molecular formula	Theoretical mass	Measured ion	Suggested ion	Fragments and adducts		ANOVA	ML PLSDA	PLSDA	Excretion kinetics
D01	0.98	Valyl-Proline^III^	C_10_H_18_N_2_O_3_	215.1390	215.1392	[M+H]^+^	197.0050	[M+H-H_2_O]^+^	*	*		A
							169.0110	[M+H-HCOOH]^+^				
							128.0170					
							100.0750	[C_5_H_10_NO]^+^				
							72.0810	[C_4_H_10_N]^+^				
D02	1.94	Isoleucyl/Leucyl-Proline^II^	C_11_H_20_N_2_O_3_	228.1474	229.1552	[M+H]^+^	183.1120	[M+H-HCOOH]^+^	*	*	*	B
							116.0700	[Pro+H]^+^				
							86.0960	[Ile/Leu+H-HCOOH]^+^				
							70.0650	[Pro+H-HCOOH]^+^				
D03	1.90	Pyroglutamyl-proline^I^	C_10_H_16_N_3_O_3_	226.1192	227.1029	[M+H]^+^	209.0920	[M-H_2_O+H]^+^	*	*		D
							181.0970	[M-HCOOH+H]^+^				
							116.070	[Pro+H]^+^				
							70.0650	[Pro-HCOOH+H]^+^				
D04	2.96	4-hydroxyphenylacetic acid^I^	C_8_H_8_O_3_	152.0473	151.0388	[M-H]^−^			*	*		A
D05	2.39	Hydroxyphenylacetic acid sulfate^II^	C_8_H_8_O_6_S	232.0042	230.9959	[M-H]^−^	187.0010	[M-H-CO_2_]^−^	*	*	*	B
							151.0380	[M-H-SO_3_]^−^				
							107.0690	[M-H-SO_3_-CO_2_]^−^				
							105.0330	[M-H-SO_3_-HCOOH]^−^				
							95.0500	[C_6_H_7_O]^−^				
							93.0320	[C_6_H_5_O]^−^				
							79.0500	[C_6_H_7_]^−^				
							77.0390	[C_6_H_5_]^−^				
D06	3.37	Phenylacetyl-glycine^I^	C_10_H_11_NO_3_	193.0739	192.0653	[M-H]^-^	148.0770	[M-CO_2_-H]^−^	*	*	*	A
							77.0400	[C_6_H_5_]^−^				
							74.0250	[Gly-H]^−^				
D07	1.93	Hydroxyphenylacetyl-glutamine^II^	C_13_H_16_N_2_O_5_	280.1059	279.0974	[M-H]^−^	261.0880	[M-H_2_O-H]^−^	*	*		B
							235.0980	[M-CO_2_-H]^−^				
							218.0810	[M-CO_2_-H_2_O-H]^-^				
							145.0600	[Glutamine-H]^−^				
							133.0290	[hydroxyphenylacetic acid -OH-H]^−^				
							127.0490	[Glutamine-H-OH]^−^				
							109.0390	[Glutamine-H-2OH]^−^				
D08	0.91	Tyramine sulfate^I^	C_8_H_11_NO_4_S	217.0409	218.0504	[M+H]^+^			*	*	*	B
D09	3.48	Phenyllactic acid^I^	C_9_H_10_O_3_	166.0630	165.0542	[M-H]^−^			*	*	*	A
D10	3.73	4-ethylphenyl sulfate^I^	C_8_H_10_O_4_S	202.0300	20.10215	[M-H]^−^	121.0640	[M-H-SO_3_]^−^	*	*	*	C
							106.0410	[M-H-SO_3_-CH_3_]^−^				
							79.9570	[SO_3_]^−^				
D11	1.78	8-Acetamido-caprylic acid^III^	C_10_H_19_NO_3_	201.1365	202.1436	[M+H]^+^	184.1340	[M+H-H_2_O]^+^	*	*	*	B
							156.1380	[M+H-HCOOH]^+^				
							138.1280	[M+H-HCOOH-H_2_O]^+^				
							128.1430	[M+H-HCOOH-CO]^+^				
							114.0920	[M+H-HCOOH-C_3_H_6_]^+^				
							100.0770	[M+H-HCOOH-C_4_H_8_]^+^				
							96.0810	[M+H-HCOOH-C_3_H_6-_H_2_O]^+^				
							84.0800	[M+H-HCOOH-C_4_H_8_-H_2_O]^+^ or [C_5_H_10_N]^+^				
							82.0650	[C_5_H_8_N]^+^				
							72.0800	[M+H-HCOOH-C_4_H_8_-CO]^+^ or [C_4_H_10_N]^+^				
							70.0650	[C_4_H_8_N]^+^				
							55.0540	[C_4_H_7_]^+^				
D12	2.65	Pyrrolidine-conjugate^III^	C_13_H_17_NO_3_	235.1208	236.1258	[M+H]^+^	190.1220	[M+H-HCOOH]^+^	*	*	*	B
							172.1120	[M+H-HCOOH-H_2_O]^+^				
							157.0892	[M+H-HCOOH-CH_3_-H_2_O]^+^				
							119.0730	[M+H-HCOOH-pyrrolidine]^+^				
							105.0702	[C_8_H_9_]^+^				
							103.0540	[C_8_H_7_]^+^				
							91.0550	[C_7_H_7_]^+^				
							79.0640	[C_6_H_7_]^+^				
							70.0650	[pyrroline+H]^+^				
D13	2.67	Pyrrolidine-conjugate^III^	C_13_H_17_NO_3_	235.1208	236.1268	[M+H]^+^	190.1220	[M+H-HCOOH]^+^	*	*	*	B
							172.1120	[M+H-HCOOH-H_2_O]^+^				
							157.0892	[M+H-HCOOH-CH_3_-H_2_O]^+^				
							119.0730	[M+H-HCOOH-pyrrolidine]^+^				
							105.0702	[C_8_H_9_]^+^				
							103.0540	[C_8_H_7_]^+^				
							91.0550	[C_7_H_7_]^+^				
							79.0640	[C_6_H_7_]^+^				
							70.0650	[pyrroline+H]^+^				
D14	3.50	Pyrrolidine-conjugate^III^	C_18_H_26_N_2_O_4_	334.1892	335.1975	[M+H]^+^	190.1250	[C_12_H_15_NO+H]^+^	*	*	*	B
							172.1160	[C_12_H_15_NO+H-H_2_O]^+^				
							119.0750	[C_12_H_15_NO+H-HCOOH-pyrrolidine]^+^				
							91.0550	[C_7_H_6_+H]^+^				
							70.0650	[pyrroline+H]^+^				
D15	3.45	Pyrrolidine-conjugate^III^	C_18_H_26_N_2_O_4_	334.1893	335.1969	[M+H]^+^	190.1250	[C_12_H_15_NO+H]^+^	*	*	*	B
							172.1160	[C_12_H_15_NO+H-H_2_O]^+^				
							119.0750	[C_12_H_15_NO+H-HCOOH-pyrrolidine]^+^				
							91.0550	[C_7_H_6_+H]^+^				
							70.0650	[pyrroline+H]^+^				
D16	3.49	Cyclohexane carboxylic acid glycine^I^	C_9_H_15_NO_3_	185.1052	184.0965	[M-H]^−^	140.1060	[M-H-CO_2_]^−^	*	*		A
							138.0910	[M-H-HCOOH]^−^				
							74.0240	[Gly-H]^−^				
D17	3.98	Ethyl-methylphenyl sulfate^III^	C_9_H_12_O_4_S	216.0456	215.0369	[M-H]^−^	135.0810	[M-H-SO_3_]^−^	*	*	*	B
							106.0420	[M-H-SO_3_-C_2_H_5_]^−^				
							79.9570	[SO_3_]^−^				
D18	0.69	unknown^IV^			259.1672	[M+H]^+^			*	*	*	B
D19	1.40	unknown^IV^	C_14_H_17_N_3_O_4_S	32.3094	324.1025	[M+H]^+^	307.0760	[M+H-NH_3_]^+^	*		*	B
							203.0880					
							185.0690					
							174.0380	[M+H-NH_3_^−^C_3_H_5_NO_2_^−^HCOOH]^+^				
							157.0760					
							130.0660					
D20	2.84	unknown^IV^	C_13_H_19_NO_3_	237.1365	238.1441	[M+H]^+^	162.0570	[C_9_H_8_NO_2_]^+^	*	*	*	B
							138.0960					
							120.0470	[C_7_H_6_NO]^+^				
							92.0510	[C_6_H_6_N]^+^				
D21	1.66	Isobutyryl-glycine^I^	C_6_H_11_NO_3_	145.0739	144.0655	[M-H]^−^	74.0245	[Glycine-H]^−^				-
D22	2.73	Isovaleryl-glycine^I^	C_7_H_13_NO_3_	159.0895	158.0809	[M-H]^−^	74.0245	[Glycine-H]^−^	*			A
D23	2.60	Angelyl-glycine^III^	C_7_H_11_NO_3_	157.0739	156.0653	[M-H]^−^	74.0245	[Glycine-H]^−^	*			A
D24	2.65	Tiglyl-glycine^I^	C_7_H_11_NO_3_	157.0739	156.0656	[M-H]^−^	74.0245	[Glycine-H]^−^	*			A

**TABLE 3:** Microbial metabolites reported with their retention time (RT), putative identity and relative identification confidence level in apex, molecular formula, theoretical neutral mass, measured precursor ion mas, measured fragments and adduct ion masses, significance in the applied statistical methods, and excretion kinetics.

Feature	RT	Metabolite (level identification)	Molecular formula	Theoretical mass	Measured ion	Suggested ion	Fragments and adducts		ANOVA	ML PLSDA	PLSDA	Excretion kinetics
MB1	2.57	Phenol sulfate^I^	C_6_H_6_O_4_S	173.9987	172.9900	[M-H]^−^	93.0339	[M-H-SO_3_]^−^				D
MB2	3.43	p-Cresol sulfate^I^	C_7_H_8_O_4_S	188.0143	187.0054	[M-H]^−^	107.0494	[M-H-SO_3_]^−^				D
MB3	3.44	p-Cresol glucuronide^I^	C_13_H_16_O_7_	284.0896	283.0834	[M-H]^−^						D
MB4	2.80	Indoxyl sulfate^III^	C_8_H_7_NO_4_S	213.0096	212.0009	[M-H]^−^	132.0455	[M-H-SO_3_]^−^				D
MB5	3.20	3-Indoxyl sulfate^I^	C_8_H_7_NO_4_S	213.0096	212.0009	[M-H]^−^	132.0455	[M-H-SO_3_]^−^				-
MB6	2.89	3-indoxyl glucuronide^I^	C_14_H_15_NO_7_	309.0849	308.0771	[M-H]^−^						D
MB7	3.51	Indolelactic acid^I^	C_11_H_11_NO_3_	205.0739	204.0655	[M-H]^−^						A
MB8	3.14	Phenylacetyl- glutamine^I^	C_13_H_16_N_2_O_4_	264.1110	263.1024	[M-H]^−^	145.0613	[Glutamine-H]^-^	*			-

### 3.3 Elimination kinetics

**FIGURE 2:**
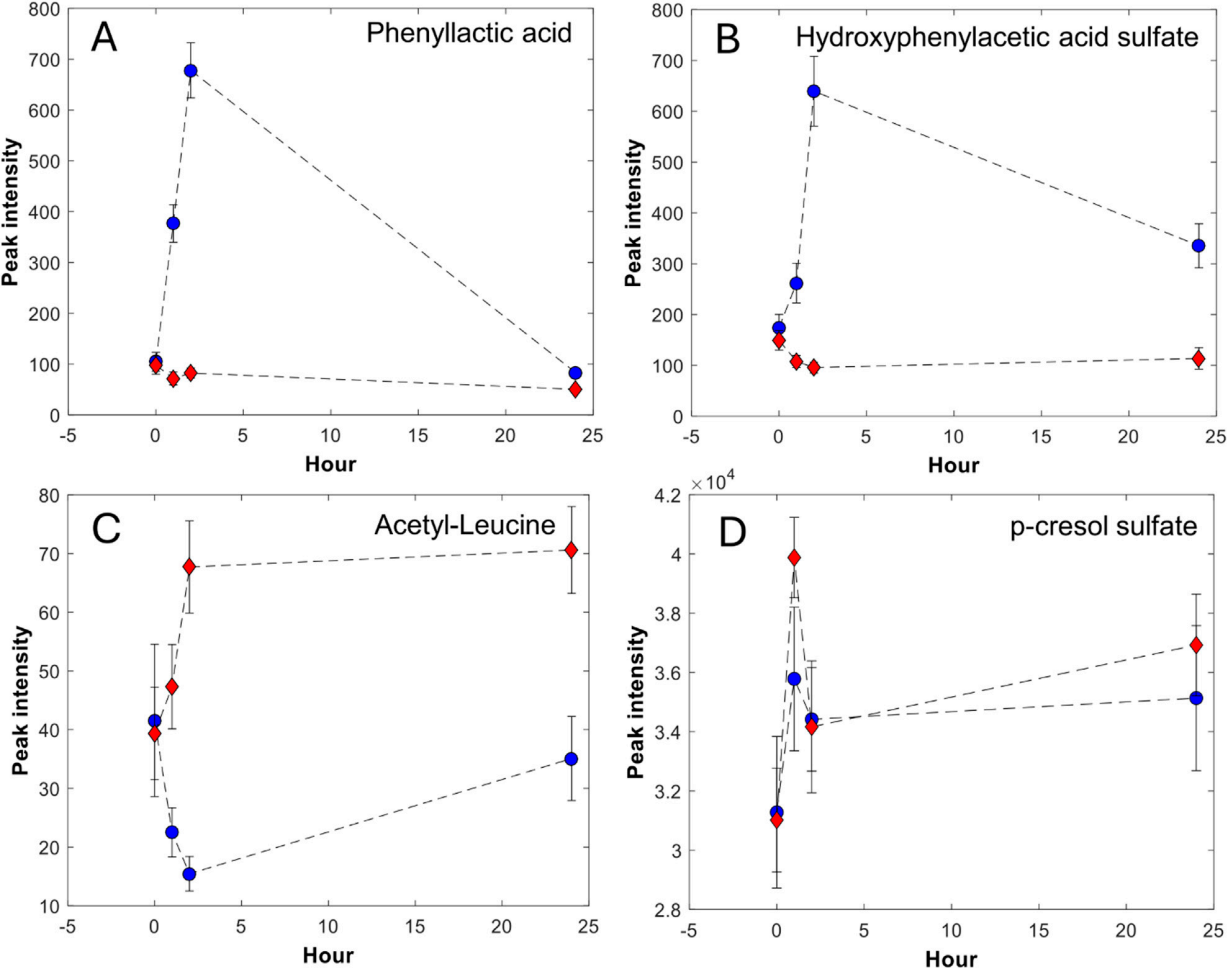
Selected metabolites categorized in the four different elimination kinetics trajectories **(A–D)**. The data points represent the mean and the bars represent the standard error of the mean; red diamond-meat, blue circle-dairy.

Based on the postprandial sampling, we report elimination kinetics trajectories of metabolites over the 24 h following the meals. We define four different kinetic trajectories, as shown in Figure 2:

A. Metabolites with fast elimination that peak at 2 or 4 h and return to baseline after 24 h.
B. Metabolites with fast elimination that peak at 4 h and then decrease, but do not return to baseline at 24 h.
C. Metabolites with moderately fast elimination that peak at 4 h and stay elevated at 24 h.
D. Metabolites that are unchanged or increase from 4 h to 24 h in both meals, in some cases with a different magnitude. In addition, the metabolite level is higher at 24 h than at baseline.

### 3.4 Identification

**FIGURE 3:**
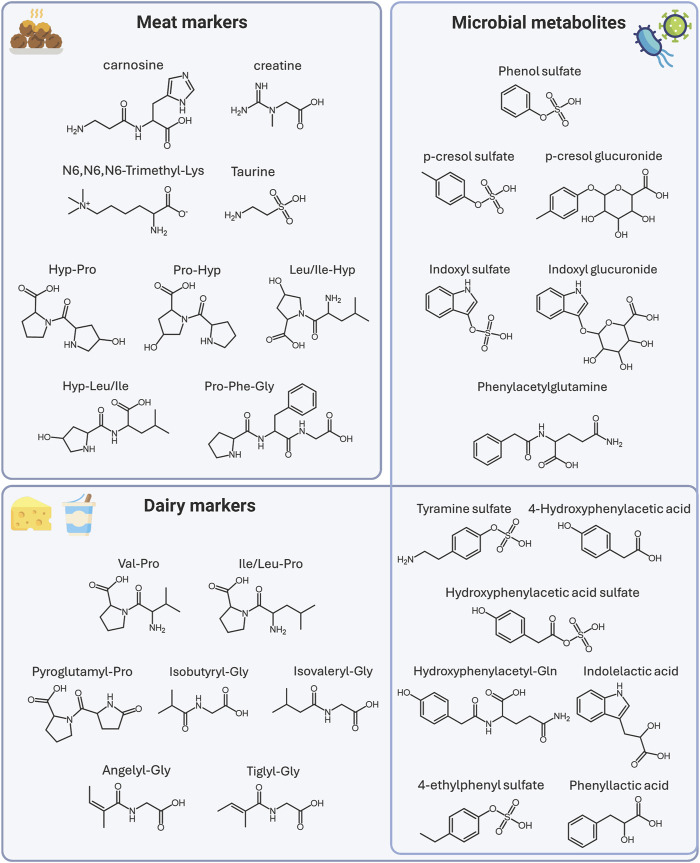
Structures of selected biomarkers of meat and dairy intake, and of proteolytic microbial metabolites. Abbreviations: Lysine (Lys), Hydroxyproline (Hyp), Proline (Pro), Phenylalanine (Phe), Valine (Val), Leucine (Leu), Isoleucine (Ile), Glycine (Gly), Glutamine (Gln). Created with BioRender.com.

Out of 50 metabolites, 21 have been confirmed with reference standards and thus identified at level I, and 29 have been tentatively identified at different levels of confidence. Information related to the identity of the metabolites, their confidence level, the theoretical and measured mass, and their fragmentation is also reported in Tables 1–3. Details on the interpretation of the MS/MS spectra have been provided in the Supplementary Material. Figure 3 reports the structure of selected meat biomarkers, dairy biomarkers, and microbial metabolites.

#### 3.4.1 Meat biomarkers

Carnosine (M01), taurine (M02), creatine (M03), and N6,N6,N6-Trimethyl-L-lysine (M04) were identified at level I. A range of di- and tripeptides, namely, hydroxyprolyl-proline (M05), prolyl-hydroxyproline (M06), leucyl/isoleucyl-hydroxyproline (M07), hydroxyprolyl-leucine/isoleucine (M08), prolyl-phenylalanyl-glycine (M09) have been tentatively identified and correlate with each other. The two isomeric dipeptides M05 and M06 show different kinetics trajectories and also partially correlate with different metabolites. The chromatographic peaks for M07 and M08 are very broad, indicating coelution of several isomers (hydroxyprolyl-leucine, leucyl-hydroxyproline, isoleucyl-hydroxyproline, hydroxyproline-isoleucine). A modified amino acid tentatively identified as acetyl-leucine (M10), does not correlate with the previously mentioned peptides but with markers M11-M17, indicating a common metabolic pattern. M11 was tentatively identified as a dihydroxybenzoic acid methyl ester and M15 as a derivative of indoleacetic acid M15. All the identified meat BFIs showed elimination kinetics categorized into trajectories A, B, or C (Supplementary Figure S4), except for N6, N6, N6-trimethyl-L-lysine (M04), which has been classified as kinetic trajectory D meaning that after 4 h it increased until 24 h for both dairy and meat, yet significantly higher after the meat intervention. The previously identified meat BFIs anserine and 3-methylhistidine were not retrieved in this data.

#### 3.4.2 Dairy biomarkers

Seven markers of dairy intake have been identified at level I, namely, pyroglutamine proline (D03), 4-hydroxyphenylacetic acid (D04), phenylacetyl-glycine (D06), tyramine sulfate (D08), phenyllactic acid (D09), 4-ethylphenyl sulfate (D10), and cyclohexanecarboxylic acid glycine (D16). The rest of 14 metabolites were only tentatively identified and belong to different classes of compounds. The first class, including markers D01 and D02, likely represents dipeptides. Marker D01 was tentatively identified as valyl-proline whereas marker D02 seems like a mixture of prolyl-leucine, prolyl-isoleucine, leucyl-proline, and isoleucyl-proline which fits both with the observed chromatographic peak broadening and the fragmentation patterns.

Next, we tentatively identified a series of pyrrolidine-based structures, i.e., D12, D13, D14, and D15. The suggested structures for D12 and D13 include pyrrolidine-ethyl-hydroxy benzoic acid. D14 and D15 are likely conjugates of D12 and D13, with a mass difference associated with piperidone.

Other markers have been tentatively identified as hydroxyphenylacetic acid sulfate (D05), hydroxyphenylacetyl-glutamine (D07), 8-acetamidocaprylic acid (D11), and ethyl-methylphenyl sulfate (D17). The rest of the markers in Table 2 are referred to as unknown because no hypothesis on their identity could be formulated.

Metabolites D01, D02, D08-D15, and D17-D20 strongly correlate with each other with a coefficient higher than 0.7 indicating a common metabolic pattern. All the newly identified dairy BFIs showed elimination kinetics categorized into trajectories A, B, or C, besides pyroglutamyl-proline (D03) which is assigned to level D because it increases also in meat at 24 h (Supplementary Figure S5).

The previously identified dairy BFIs galactose, lactose, galacticol, galactonate, and isovalerylglutamic acid were not retrieved from our data. The acyl-glycines isobutyryl-glycine (D21), isovaleryl-glycine (D22), and tiglyl-glycine (D24) were found and identified at level I, together with an isomer of tiglyl-glycine, namely, angelyl-glycine (D23), identified at level II. All these BFIs except for isobutyryl-glycine were selected by ANOVA and they all showed kinetics trajectory A (Supplementary Figure S5). The acyl-glycines correlate with each other but not with the rest of the markers.

#### 3.4.3 Microbial metabolites

Phenol sulfate (MB1), p-cresol sulfate (MB2) and glucuronide (MB3), 3-indoxyl sulfate (MB5) and glucuronide (MB6), indolelactic acid (MB7) and phenylacetyl-glutamine (MB8) were extracted from the raw dataset based on the RT and *m/z* of corresponding standards (level I). An isomer of 3-indoxyl sulfate (MB4) was identified at level II. All the microbial metabolites except for 3-indoxyl sulfate, phenylacetyl-glutamine and indolelactic acid were categorized as kinetics trajectory D (Supplementary Figure S6). These metabolites had an acute increase at 0–2 h, followed by a decrease at 2–4 h, but in the 4–24 h sample their levels were higher than the baseline levels, and there was no difference between the meat and dairy meals. Phenylacetyl-glutamine and 3-indoxyl sulfate showed this same trend for the meat intervention group but not for the dairy, and cannot be then classified as trajectory D. However, while phenylacetyl-glutamine showed no significant difference between dairy and meat at any time point, 3-indoxyl sulfate showed higher levels in meat compared to dairy at 2 h. The elimination kinetic profile of indolelactic acid shows that this microbial metabolite behaves like a postprandial BFI for dairy even though it was not selected by univariate data analysis, because significantly different between the two meals only at 1 time point. In addition, it does not correlate with the other microbial metabolites. p-cresol glucuronide (MB3) shows an inverse correlation with all the other microbial metabolites and with the dairy markers. Finally, among the dairy markers, 4-ethylphenyl sulfate (D10) and the acyl-glycines D21-D24 partially correlate with most of the microbial metabolites (r = 0.5). Phenylacetic acid, phenol glucuronide, tyramine, indolepropionic acid, and indoleacetic acid could not be retrieved in the raw data.

## 4 Discussion

The primary aim of this work was to highlight BFIs that differentiate between a meal with meat or dairy consumed without prior dietary restrictions, and to validate previously reported BFIs of the two foods. By inspecting the elimination kinetics of the different biomarkers, we provided a set of BFIs for meat and dairy intake with fast (<24-h) or relatively fast (>24-h) time windows of excretion. As a secondary aim, we explored whether previously reported proteolytic microbial metabolites differ after intake of meat or dairy.

### 4.1 Meat intake biomarkers

Carnosine (M01), taurine (M02) and creatine (M03), previously reported meat biomarkers (Cuparencu et al., 2019a), were confirmed here without a dietary restriction of meat intake during the previous day. Taurine is usually considered unspecific due to its similar contents in dairy products (Cuparencu et al., 2019b), but here we observe a higher elimination rate after meat, suggesting that its inclusion in combined biomarkers panels to differentiate between the two sources may prove valuable.

Six hydroxyproline (Hyp) containing dipeptides, i.e., Pro-Hyp, Hyp-Pro, Ile/Leu-Hyp, Hyp-Ile/Leu, originating from the collagen helix were also reconfirmed (Cuparencu et al., 2019a). However, these dipeptides were not reported among the best predictors of meat intake in a previous study (Cuparencu et al., 2019b) pointing at their lack of robustness as meat BFIs.

Anserine and 3-methylhistidine were found as very low intensity and noisy peaks in the data, possibly due to the elution of the peaks in the dead volume of the column, causing high ion suppression. In addition, anserine is mainly found in chicken and therefore studies of red meat provide a limited exposure to this metabolite. Anserine is a precursor of 3-methylhistidine, and while we see a peak matching the mass and RTs of the methyl-histidines, i.e.,1-methyl histidine (1-MH or N^τ^-MH) and 3-methyl histidine (3-MH or N^π^-MH), our chromatographic method could not distinguish between 1-MH and 3-MH in this study. Based on the kinetic profile of the feature (data not shown), it is most probable that it corresponds to 1-MH, a marker that is not robust for meat intake due to its endogenous production and high individual variability (Cuparencu et al., 2019a). A similar challenge is usually seen for carnitine that is often reported as a meat BFI in experimental and observational studies, even though levels vary between individuals due to its endogenous function in amino acid and lipid catabolism. Here, the carnitine precursor, N6,N6,N6-trimethyl-L-lysine (M04), was observed as a BFI peaking >4 h after the meat meal and therefore with a longer time window. Previously reported after intake of fish and meat (Cuparencu et al., 2019b), we speculate that its presence in meat histones delays liberation, causing the late appearance. The kinetics observed here indicate large intestinal liberation, i.e., following microbial protein degradation. A few other markers observed after red meat intake cannot be identified with confidence. A derivative of indoleacetic acid (M15) might derive from microbial proteolytic metabolism.

### 4.2 Dairy intake biomarkers

We identified a range of proline-dipeptides as dairy BFIs. Isoleucyl-proline was previously reported as an intake biomarker of caseinoglyco-macropeptide (Stanstrup et al., 2014b), while pyroglutamyl-proline has also been found as biomarker of whey hydrolysate intake (Stanstrup et al., 2014a). Proline dipeptide levels depend on prolidase for degradation and liberation of proline, and this same enzyme is also needed to degrade the hydroxyproline-dipeptides observed after meat intake (Eni-Aganga et al., 2021). The observation of these dipeptides in urine suggests an insufficient activity of prolidase. It is conceivable that the fast liberation of the dipeptides after dairy or meat intake overwhelms the prolinase capacity in the intestine, increasing their absorption and excretion into urine. Among the proline dipeptides pyroglutamyl-proline is the only one that increases after meat intake at 24 h. This might be due to the presence of such compound also in other types of foods such as wheat gluten (Schlichtherle-Cerny and Amadò, 2002).

4-hydroxyphenyl acetic acid along with its sulfate and glutamine conjugates, as well as tyramine sulfate, phenyllactic acid, and 4-ethylphenyl sulfate are likely deriving from microbial fermentation in the cheese. In a previous work, both tyramine and 4-hydroxyphenylacetic acid were found as cheese urinary biomarkers. These metabolites were supposed to be formed by microbial metabolism during cheese ripening (Hjerpsted et al., 2014) and further conjugated into their sulfate and glutamine conjugates by the host secondary metabolism. Phenyllactic acid and 4-ethylphenyl sulfate are two commonly reported gut microbial metabolites (Zheng et al., 2021). However, while 4-ethylphenyl sulfate has only been shown to derive from proteolytic fermentation in the human gut, phenyllactic acid can also derive from cheese fermentation (Valerio et al., 2004). Phenyllactic acid, as well as tyramine sulfate, 4-hydroxyphenylacetic acid and its derivatives, show a fast elimination kinetics, suggesting that the metabolites are already present in the cheese product. In contrast, the slower excretion of 4-ethylphenyl sulfate, together with a positive correlation with the gut microbial metabolites, might suggest that this metabolite is a product of the gut microbiota after intake of specific dairy-derived proteins. Nevertheless, since 4-ethylphenyl sulfate derives from phenylalanine, which is in both dairy and meat, the higher levels after dairy intake might be explained by the presence in the dairy of 4-ethylphenol, which is further sulfated by the host metabolism in the following 24 h.

Cyclohexane carboxylic acid glycine has been previously reported after berry intake (Cuparencu et al., 2016) and its presence as a dairy biomarker in this study points at the flavour of the yogurt provided in the dairy meal. The origin of the other urinary metabolites observed following intake of the dairy meal, namely, acetylated 8-aminocaprylic acid, ethyl-methylphenyl sulfate, and four pyrrolidine conjugates are uncertain. Since proline peptides increase after dairy intake, it would be conceivable that the pyrrolidine containing compounds observed here may derive from decarboxylated proline peptides.

Finally, several acylglycines have been identified as dairy intake biomarkers in previous studies investigating biomarkers of cheese intake (Badenhorst et al., 2013; Hjerpsted et al., 2014) and were thus confirmed here. These were not originally selected in the list of dairy biomarkers due to our strict criteria for BFIs selection and because they slightly increase also after meat intake, but all except isobutyryl-glycine were selected in the repeated measures ANOVA analysis. Isobutyryl-glycine also differs from the other acyl-glycines with respect to its kinetic profile, which is type A for dairy, but similar to the microbial metabolites type D for meat. In addition, the acyl-glycines slightly correlate with the gut microbial metabolites. Acyl-glycines might be higher in dairy, but increased after both meat and dairy, because they derive from degradation of branched-chain amino acids, which are more abundant in dairy products (Hjerpsted et al., 2014). However, they might also derive from glycine conjugation to branched-chain fatty acids, which can be present in cheese or can also originate from the proteolytic fermentation by the gut bacteria (Blachier et al., 2019).

### 4.3 Proteolytic microbial metabolites and health implications

Several microbial metabolites deriving from proteolytic fermentation are important in both health and disease (De Mello et al., 2017; Dodd et al., 2017; Roager and Dragsted, 2019). Since protein-rich products are responsible for their formation, we have targeted these metabolites to explore potential differences between red meat and dairy intake. This exploration is, however, complicated by the protein microbial fermentation occurring during dairy processing (Melkonian et al., 2023). Some of the putative BFIs observed may therefore have dual origins as processing products as well as gut fermentation products, as observed for 4-ethylphenyl sulfate. We hypothesize that microbial metabolites showing a kinetics excretion of type A, B or C are already present in the dairy product because they are excreted before the food has reached the large intestine at 3–6 h, and they are significantly different between meat and dairy, while metabolites with kinetics excretion D, which are not significantly different between dairy and meat, are produced by the human gut microbiota. All microbial metabolites except for 3-indoxyl sulfate, phenylacetyl-glutamine, and indolelactic acid belong to class D. 3-Indoxyl sulfate was shown to increase after both meals, but the signal was stronger after meat intake compared to dairy. Indoxyl sulfate is a metabolite of tryptophan. Depending on the gut environment, the presence of dietary fiber, as well as the microbiota composition, microbes can form indolepropionic acid, a health promoting metabolite (De Mello et al., 2017; Roager and Licht, 2018; Xue et al., 2022), or indoxyl sulfate, an uremic toxin reported in kidney disease (Sinha et al., 2024). The higher level following the meat meal might reflect the slightly higher level of tryptophan in red meats. However, the different trend in the excretion after dairy intake, which is more similar to the other dairy BFIs than to the microbial metabolites suggests that indole or even 3-indoxyl sulfate might be already present in the dairy product. In similarity, phenylacetyl-glutamine shows a different elimination trend following dairy and meat, although there is no significant difference in excretion between the two diets. The levels of 3-indoxyl sulfate and phenylacetyl-glutamine might therefore derive from a combination of fermentation in the dairy and in the gut. Further studies should clarify the source of these metabolites in urine after intake of fermented and unfermented protein-derived meals, to better understand their different roles in human health.

Lastly, indolelactic acid shows type A excretion kinetics, thereby behaving as postprandial BFI for the dairy meal in this study. This is not surprising considering that lactic acid bacteria are used as starter cultures in the fermentation of dairy products. Indolelactic acid has been previously reported as a dairy BFI (Bütikofer et al., 2022; Li et al., 2021; Pimentel et al., 2020). Although in our study indolelactic acid showed a significant time*meal interaction in repeated measures ANOVA, it was only significant at one time point, therefore it was not selected as dairy BFI by our strict criteria. However, additional time points at 6–8 h would have probably enabled the selection of this additional dairy BFI. Indole and phenyllactic acids are generally associated with anti-inflammatory and immunomodulating effects in the infant gut but health effects in adults have not been consistently observed (Laursen et al., 2021). Interestingly, p-cresol glucuronide was inversely correlated with the meat markers and with all other microbial metabolites. These results suggest a different pathway of elimination of p-cresol sulfate and p-cresol glucuronide and should be further investigated since the two conjugates may potentially have different effects on glucose metabolism and insulin resistance (Koppe et al., 2017).

### 4.4 Combined markers to improve the dietary assessment of meat and dairy intakes

Combined markers have been shown to outperform single markers in classifying intake of dairy (Li et al., 2021), meat (Cuparencu et al., 2019a), and other foods (Gürdeniz et al., 2016; Vázquez-Manjarrez et al., 2019; Xi et al., 2021), and are thus a useful strategy to overcome the lack of specificity of single BFIs. For rich-protein sources like meat and dairy, a pattern of distinct amino acid-derived metabolites could in theory serve as BFIs. However, as already demonstrated for meat, Hyp-Pro peptides are not good predictors of intake by themselves (Cuparencu et al., 2019b) and prediction studies targeting dairy intake through proline-dipeptides are lacking. To differentiate between intakes of meat and dairy through protein-derived metabolites, we suggest a ratio between proline- and hydroxyproline-peptides. These should be combined with taurine, also shown to differentiate the two protein foods in this study, and with additional biomarkers for meat and dairy, to test their prediction performance in individuals consuming their habitual diet and document parsimonious models.

Combinations of BFIs with different excretion kinetics might prove useful to determine the time since intake, as recently proposed (Cuparencu et al., 2024). For example, measuring phenyllactic acid and 4-ethylphenyl sulfate in the same urine sample would indicate that dairy has been consumed in the past 2–3 days, whereas the presence of phenyllactic acid only would indicate intake in the last 6–10 h, or possibly last 24 h if a pooled 24-h urine sample is available. We recently suggested the concept of time windows for sampling to describe the period after intake where a particular BFI can be measured to reliably detect the consumption of its corresponding food (Cuparencu et al., 2024). This approach, coupled with multisampling in observational studies (Cuparencu et al., 2024), could facilitate a better understanding of frequency of food intake through biomarkers, to complement the less reliable dietary information obtained from food frequency questionnaires.

### 4.5 Strengths and limitations

For biomarker discovery, we employed a combination of univariate (repeated measures ANOVA on time-series samples) and multivariate data analysis approaches (PLSDA and ML-PLSDA on pooled 24 h samples). This allowed us to 1) reduce false discoveries, since biomarkers were only put forward if they were selected by both univariate and at least one of the two multivariate analyses; 2) leverage the paired data structure of the cross-over design, by correcting participant-related random effects (ML-PLSDA), and 3) investigate the between-subject variation separately from the within-subject variation to better understand the effect of the correction for the random effect of the subjects (ML-PLSDA vs. PLSDA) (Westerhuis et al., 2010). ML-PLSDA enabled the selection of nine additional metabolites, that were otherwise not picked up by PLSDA, such as hydroxyprolyl-leucine/isoleucine for meat, and valyl-proline for dairy. However, some of the selected biomarkers are also produced endogenously (Cuparencu et al., 2019a) and show variability between subjects; their use as BFIs may therefore depend on additional samples from the same individual to correct for individuality and therefore require further validation in independent studies.

The postprandial sampling allowed us to describe kinetics profiles that consequently document the time window for sampling for a range of new biomarkers, i.e., sampling time <24 h after intake for metabolites with kinetics type A and B, and >24 h for type C and D.

In the current study we target very common foods, dairy and red meat, without restrictions on prior intake. This describes an additional aspect of their robustness, showing that the marker intensity after a single meal is sufficient. Usually, biomarker development occurs in highly controlled studies with 2–3 days run-in periods during which the intended test foods are avoided. Here we show that, even in a population that consumes meat frequently (data not shown), BFIs reflecting recent intake are still useful. None of the BFIs saturates with repeated intake (Dragsted et al., 2018), even though many, e.g., M06, M10, M12, M17, D03, D10, and D17 among others, show a carry-over effect from the intake of the preceding day.

The current work is limited by the lack of an independent dataset to validate our biomarker discovery findings and to test the performance of the combined BFIs suggested. Future work should address this in subjects having their habitual diets and test biomarker combinations that differentiate between meat and dairy. Another limiting factor is that we investigated the abundance of known metabolites in an untargeted run; running the same investigation using targeted analysis would likely allow measurement of the less abundant BFIs, such as anserine and 3-MH, providing a more comprehensive picture of the differences between protein-matched meals with meat or dairy. Lastly, some of the better validated single and combined dairy BFIs, for example, galactose derivatives, were not confirmed in this study likely due to the chromatography used. Previously, these metabolites have been measured by GC-MS (Li et al., 2021), whereas we employed a LC-MS platform here.

## 5 Conclusion

In this study we aimed to validate known BFIs and to find additional ones able to discriminate between dairy or meat protein meals. This was done against a background of habitual intakes to document robust BFIs. The BFIs were selected by a combination of multivariate pattern analyses and FDR-corrected univariate methods. Several well-known meat intake biomarkers were re-confirmed, including carnosine, taurine, and creatine, as well as several hydroxyproline-containing di-peptides. Dairy intake was mainly reflected by catabolites of aromatic- and branched-chain amino acids. Some of these BFIs reflect only recent intake and may only be useful as compliance markers, while others have time windows higher than 24 h, making them potentially more useful for assessing average dietary intake from pooled urine samples. Our results indicate the presence of microbial metabolites such as phenyllactic acid, tyramine sulfate, 4-ethylphenyl sulfate, and indolelactic acid already in dairy products, with potential utility as BFIs of dairy. Other microbial proteolysis products showed only limited differences between the protein sources. However, the pattern of some metabolites, such as 3-indoxyl sulfate and phenylacetyl-glutamine, suggested a dual origin from microbial metabolism in the dairy and in the gut, emphasizing the need to consider them as part of combined BFI panels. Further studies will be needed to elucidate the source of these metabolites in urine after protein-derived meals.

## Data Availability

The data presented in the study are deposited in the MassIVE repository, accession number MSV000095739.
