# Supplementary material for: Metabolomics-based biomarkers of fermented dairy and red meat intake: a randomized controlled trial in healthy adults
Source: Front Chem. 2024 Sep 24;12:1461331. doi: 10.3389/fchem.2024.1461331 (PMC11459089; doi:10.3389/fchem.2024.1461331)
Supplement: Supplementary file 1 [file Presentation1.pdf]

Supplementary Material

1 Methods

Table S1. Results from variable selection obtained for each pair of calibration set/validation set. Variable selection has been carried out 10 times (10 models) on different subsets of samples (calibration set, n=30) and the model has been validated in external sample sets (validation set, n=4). The selected variables that were present at least in 8 of the 10 different models were kept for further investigation. The Aurea Under the Curve (AUC) express the goodness of each model built on selected variables in classifying the samples of the validation set.

	PLS-DA				ML-PLSDA			
**Model #**	**Number of features selected**	**AUC for validation test**	**Class. Error before VarSel**	**Class. Error after VarSel**	**Number of features selected**	**AUC for validation test**	**Class. Error before VarSel**	**Class. Error after VarSel**
1	65	1	0.43	0	79	1	0.37	0
2	73	1	0.38	0	77	1	0.42	0
3	65	1	0.41	0	99	1	0.36	0
4	83	1	0.38	0.25	74	1	0.42	0
5	69	1	0.4	0	73	1	0.34	0
6	67	1	0.32	0	77	1	0.38	0
7	70	1	0.46	0	78	1	0.48	0
8	69	1	0.44	0	96	1	0.48	0
9	82	1	0.44	0	80	1	0.40	0
10	82	1	0.48	0	82	1	0.38	0
**Common features**	**52**				**67**			

2 Statistical analysis results

Supplementary Figure 1. Scores plot (left) and prediction plot (right) for the 52 features selected by PLSDA.

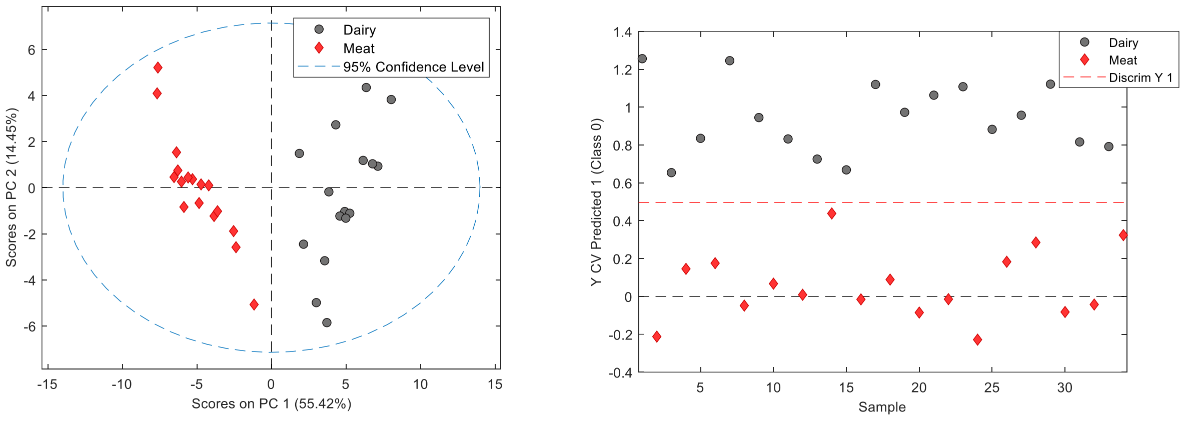

Supplementary Figure 2. Scores plot (left) and prediction plot (right) for the 67 features selected by MLPLSDA.

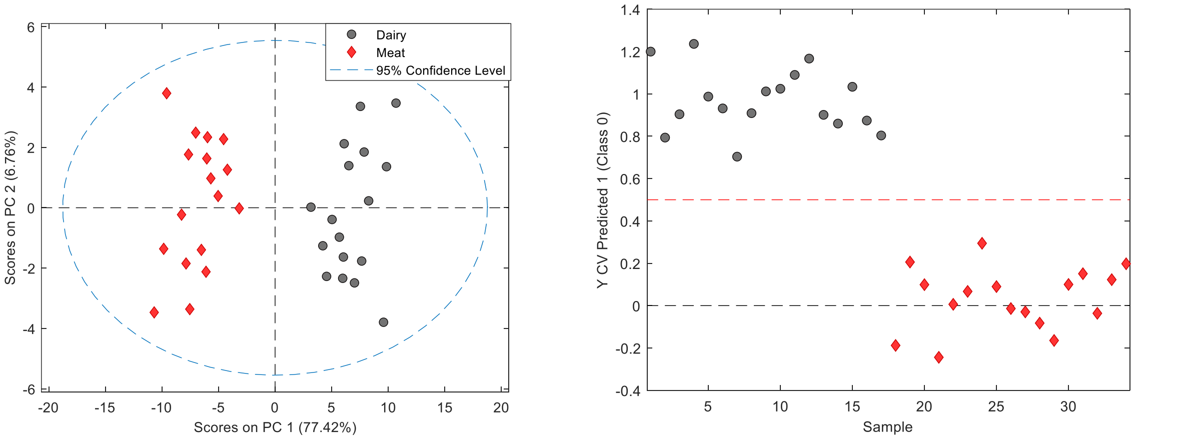

Supplementary Figure 3. Correlation map showing the Pearson correlation coefficients of newly discovered as well as targeted BFIs and microbial metabolites.

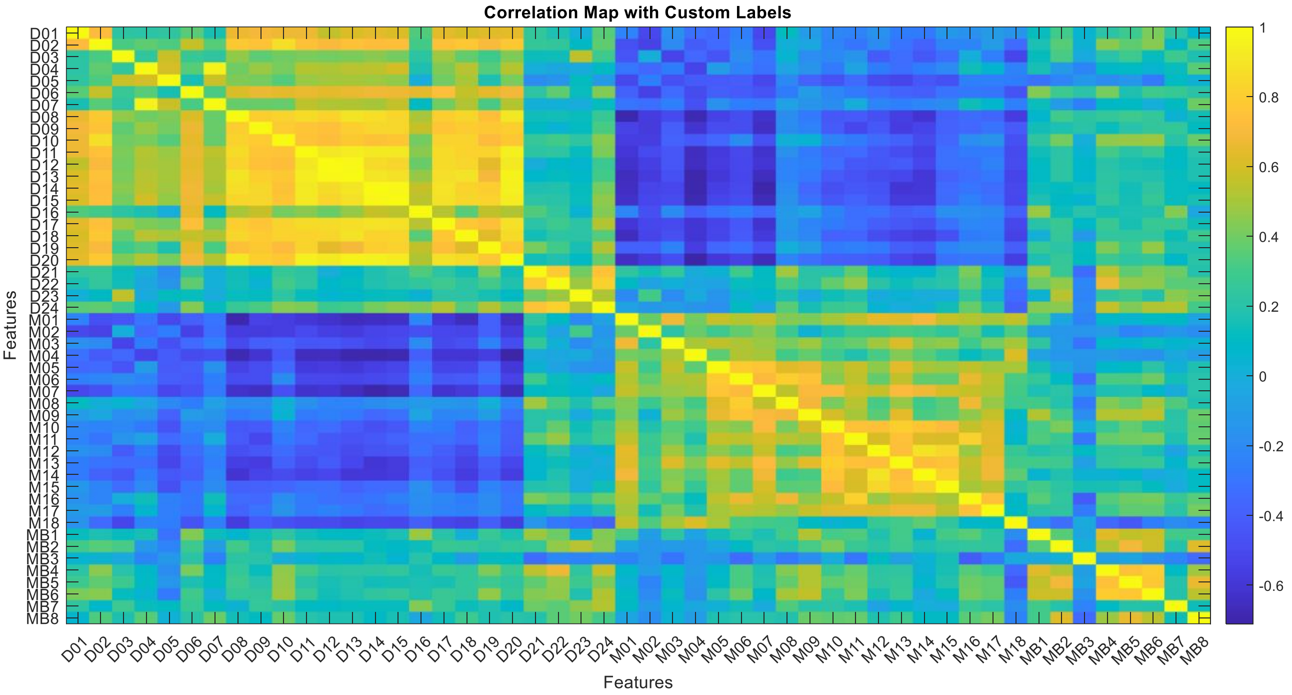

3 Kinetics curves

Supplementary Figure 4. Elimination kinetics curve for the selected meat biomarkers.

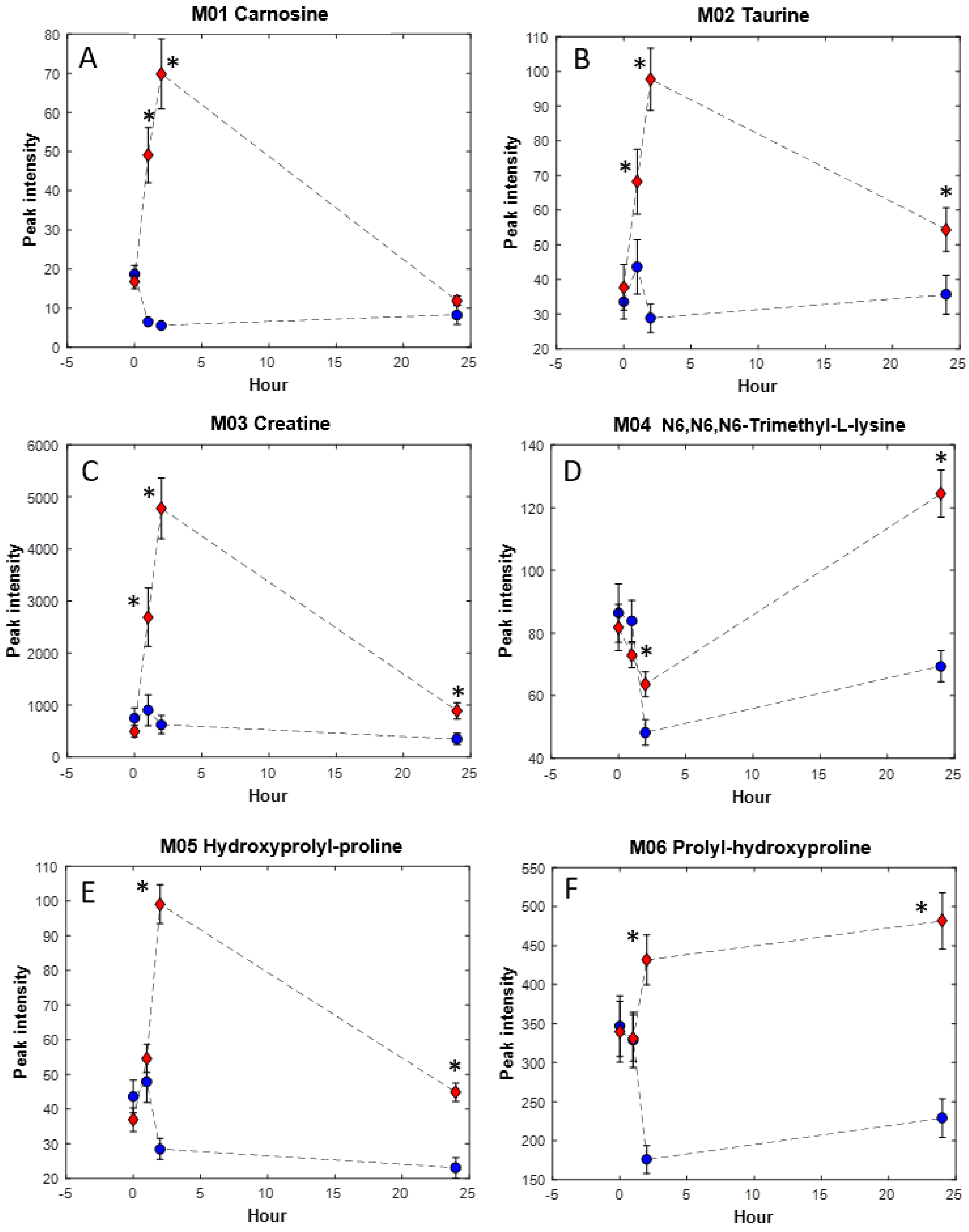

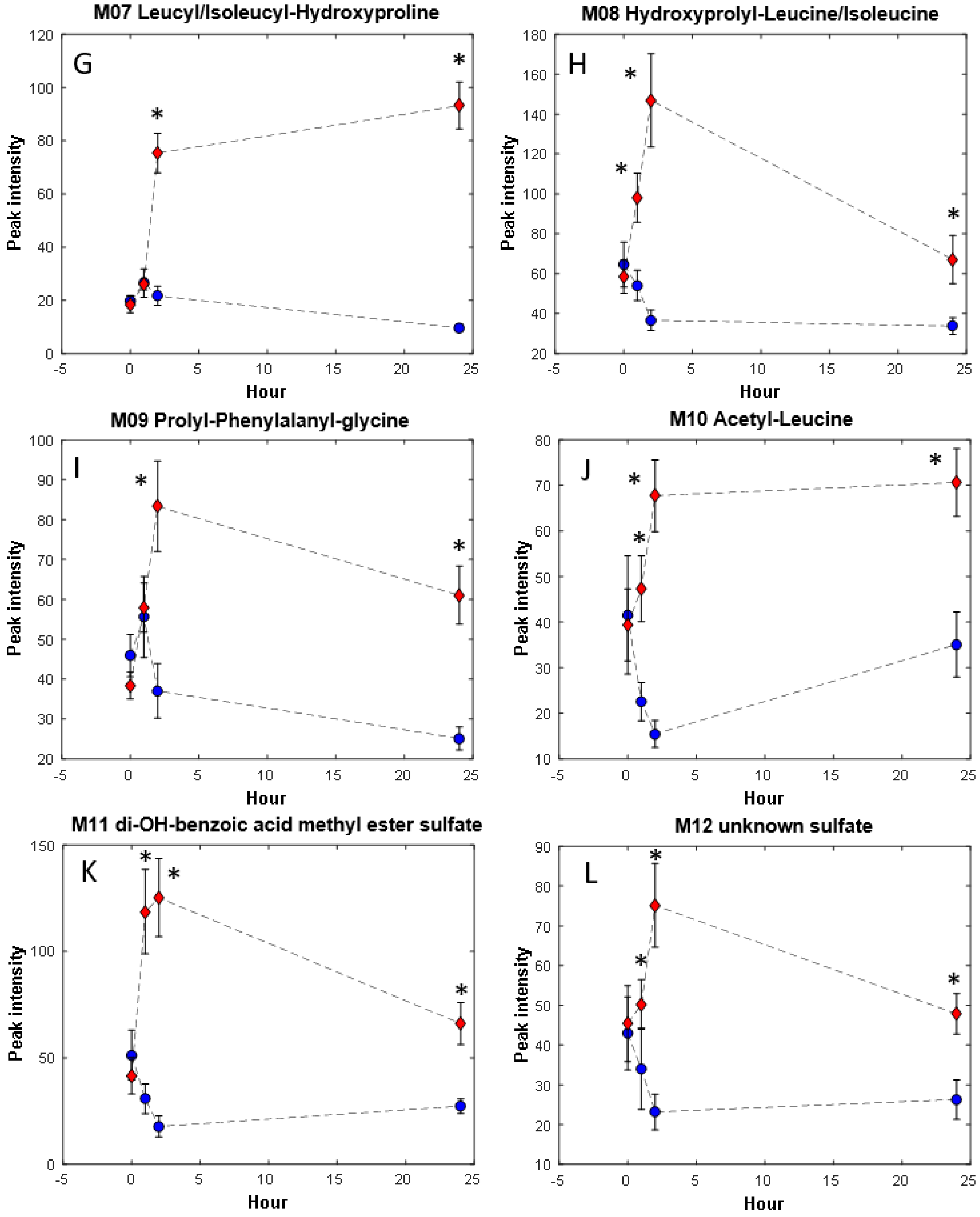
M07 Leucyl/Isoleucyl-Hydroxyproline
G
Peak intensity
Hour
M08 Hydroxyprolyl-Leucine/Isoleucine
H
Peak intensity
Hour
M09 Prolyl-Phenylalanyl-glycine
I
Peak intensity
Hour
M10 Acetyl-Leucine
J
Peak intensity
Hour
M11 di-OH-benzoic acid methyl ester sulfate
K
Peak intensity
Hour
M12 unknown sulfate
L
Peak intensity
Hour

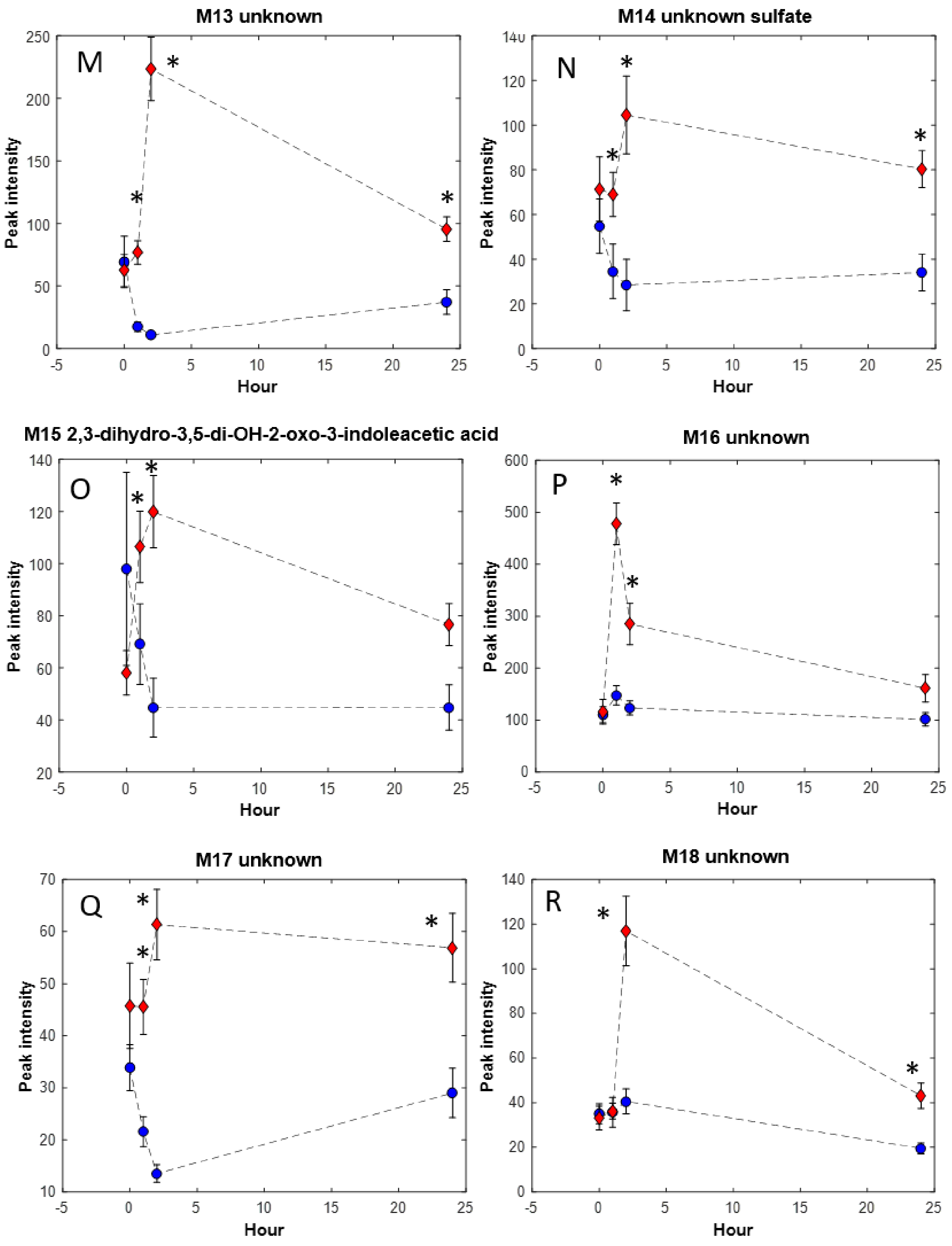
M13 unknown
M
M14 unknown sulfate
N
M15 2,3-dihydro-3,5-di-OH-2-oxo-3-indoleacetic acid
O
M16 unknown
P
M17 unknown
Q
M18 unknown
R
Peak intensity
Hour
*

Supplementary Figure 5. Elimination kinetics curve for the selected dairy biomarkers

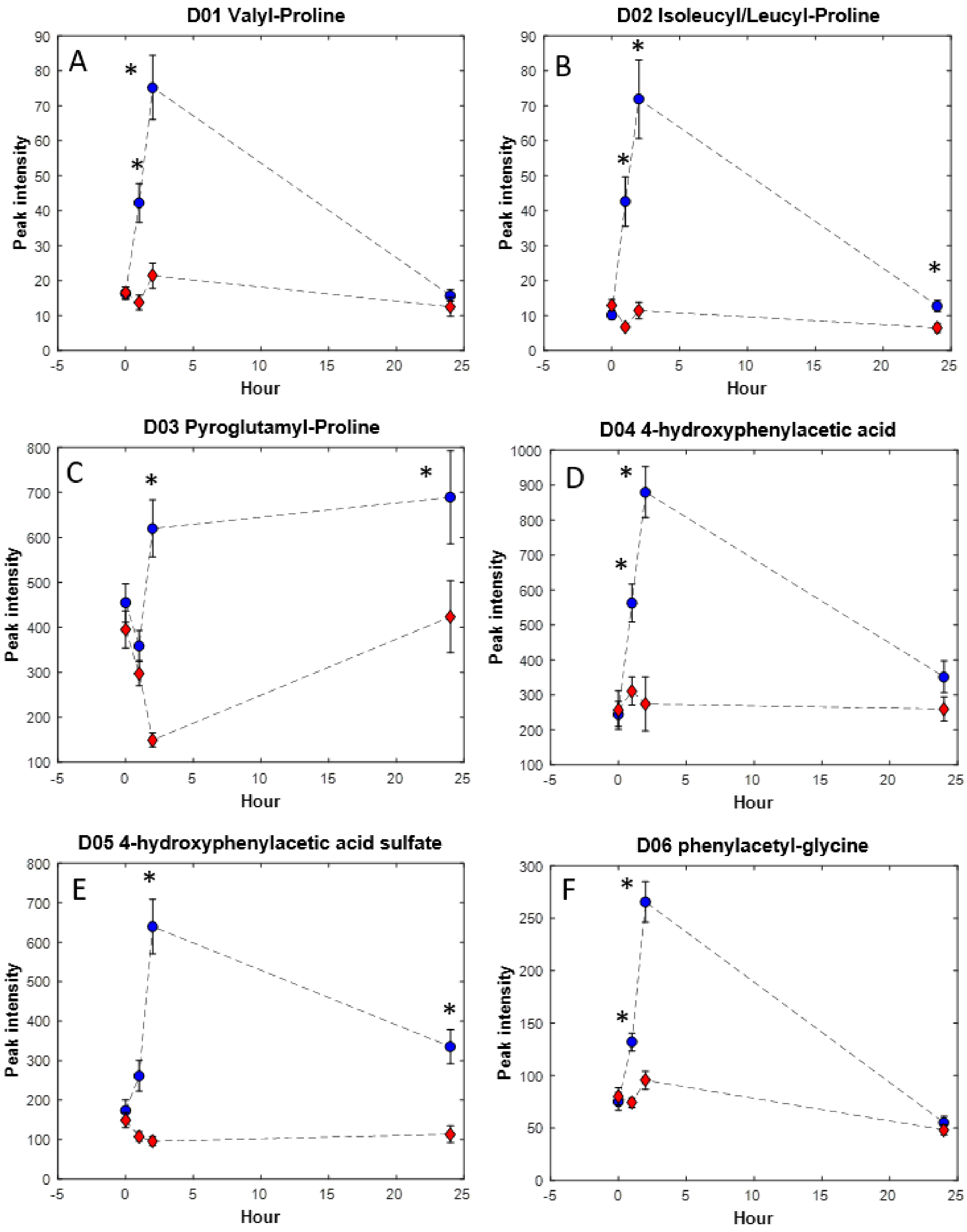

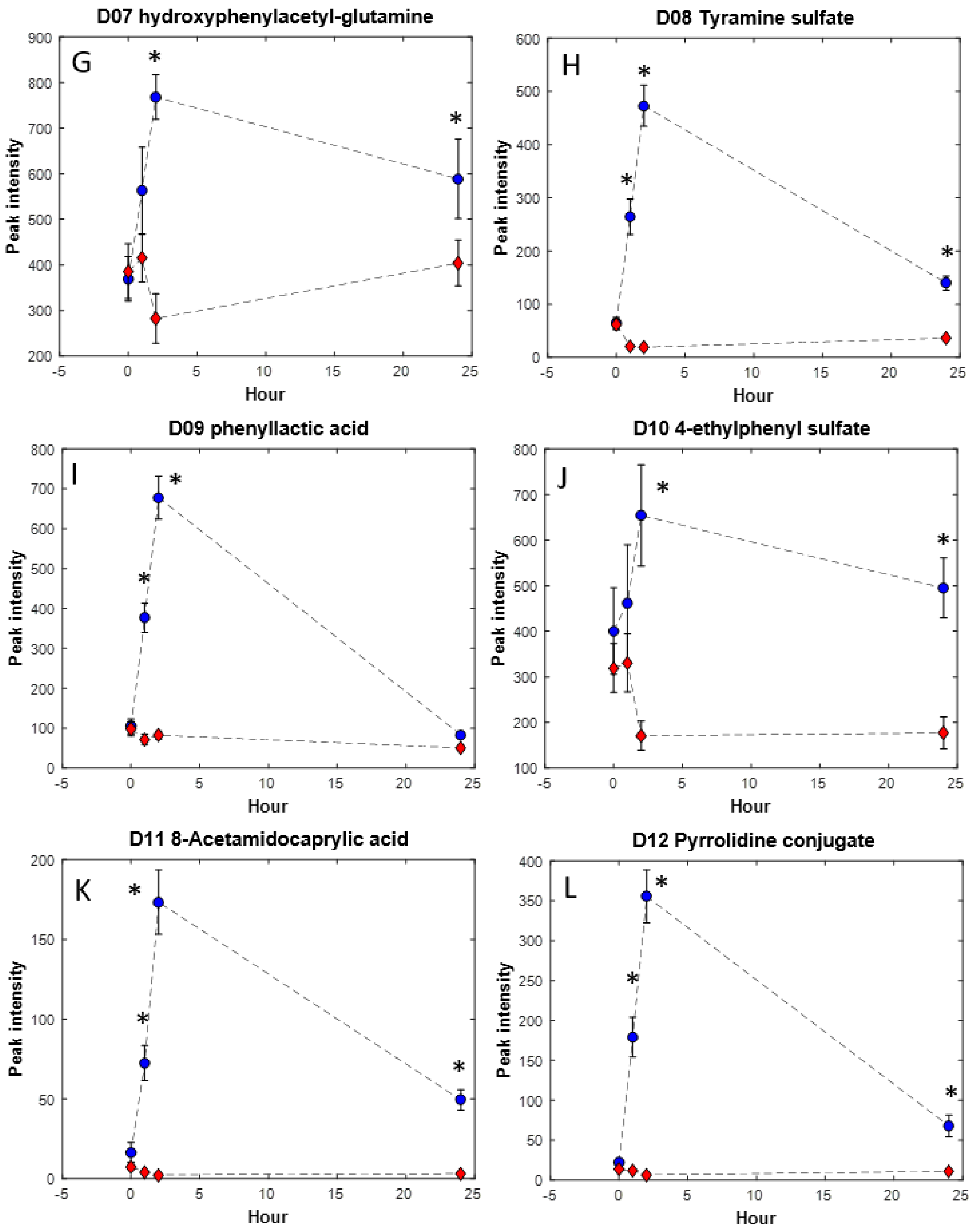
D07 hydroxyphenylacetyl-glutamine
G
Peak intensity
Hour
D08 Tyramine sulfate
H
Peak intensity
Hour
D09 phenyllactic acid
I
Peak intensity
Hour
D10 4-ethylphenyl sulfate
J
Peak intensity
Hour
D11 8-Acetamidocaprylic acid
K
Peak intensity
Hour
D12 Pyrrolidine conjugate
L
Peak intensity
Hour

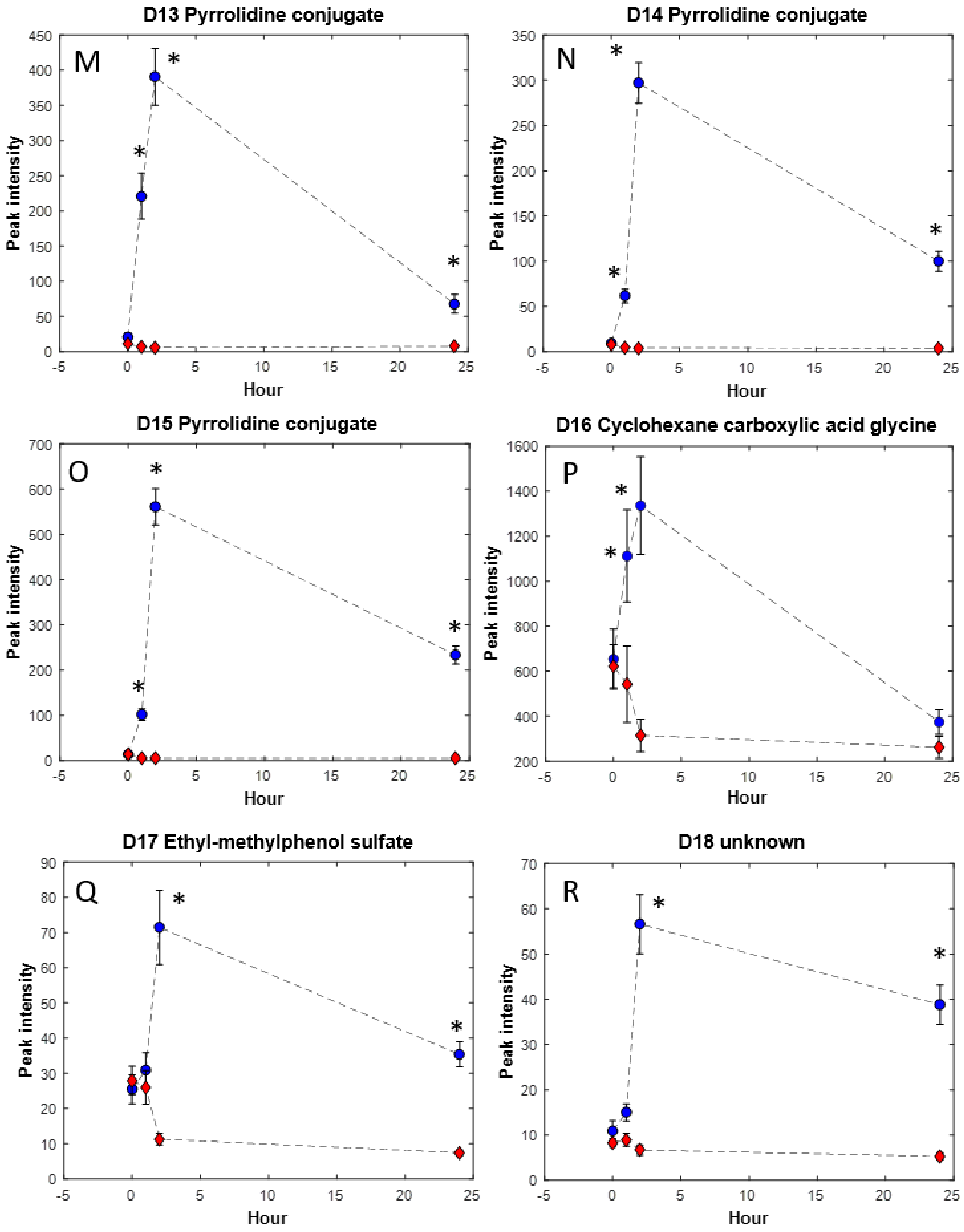
D13 Pyrrolidine conjugate
M
D14 Pyrrolidine conjugate
N
D15 Pyrrolidine conjugate
O
D16 Cyclohexane carboxylic acid glycine
P
D17 Ethyl-methylphenol sulfate
Q
D18 unknown
R
Peak intensity
Hour

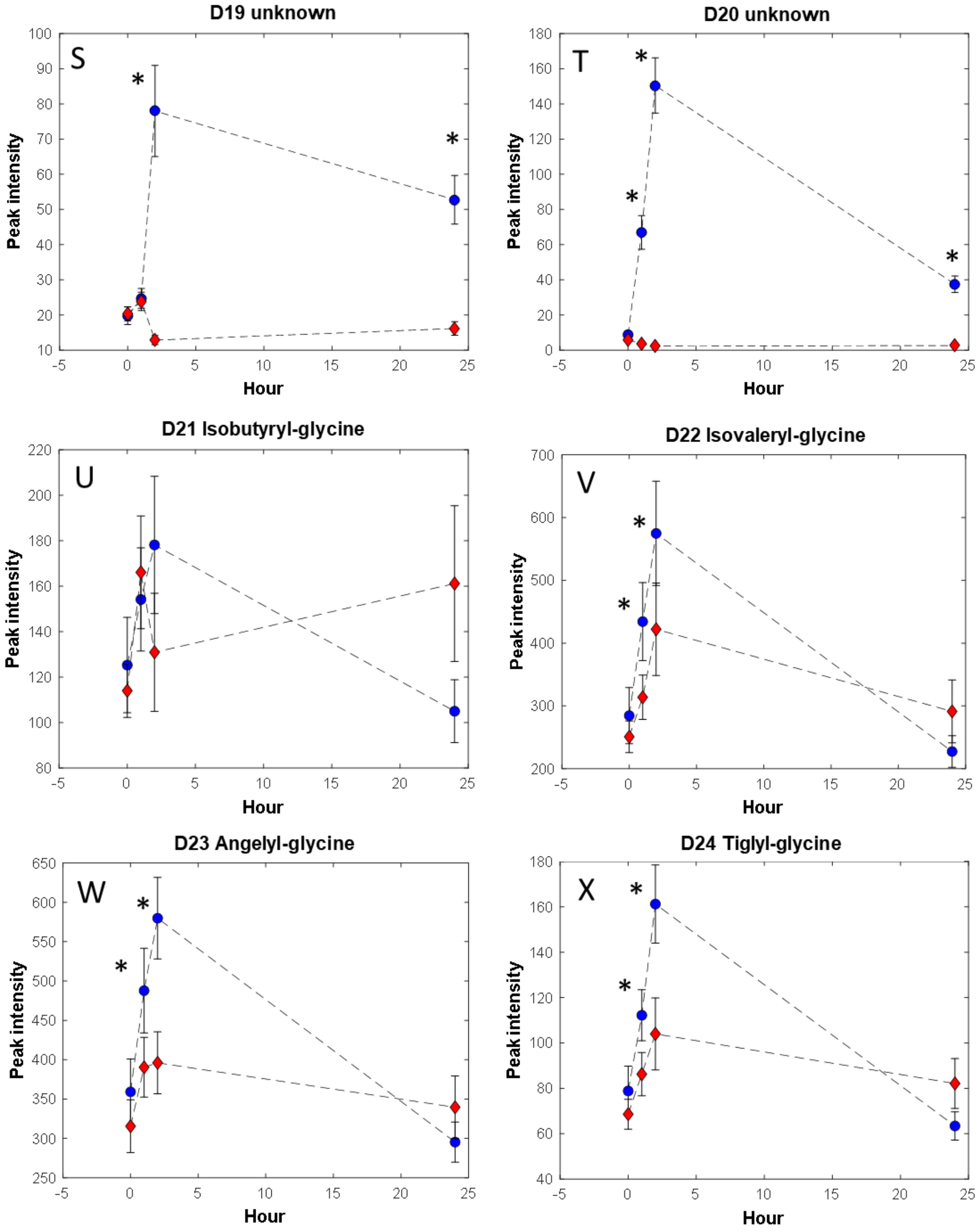
D19 unknown
S
Peak intensity
Hour
D20 unknown
T
Peak intensity
Hour
D21 Isobutyryl-glycine
U
Peak intensity
Hour
D22 Isovaleryl-glycine
V
Peak intensity
Hour
D23 Angelyl-glycine
W
Peak intensity
Hour
D24 Tiglyl-glycine
X
Peak intensity
Hour

Supplementary Figure 6. Elimination kinetics curve for the microbial metabolites.

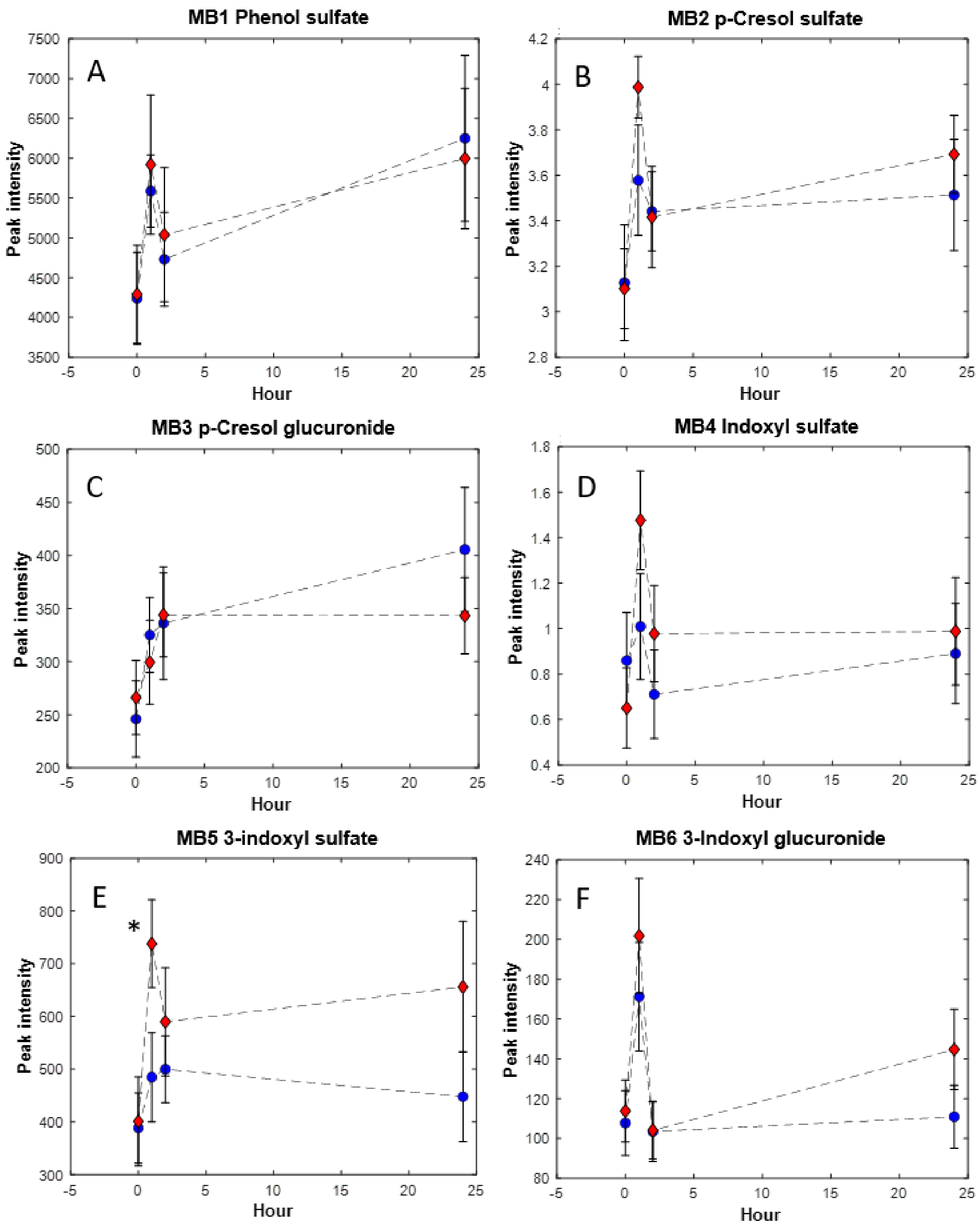

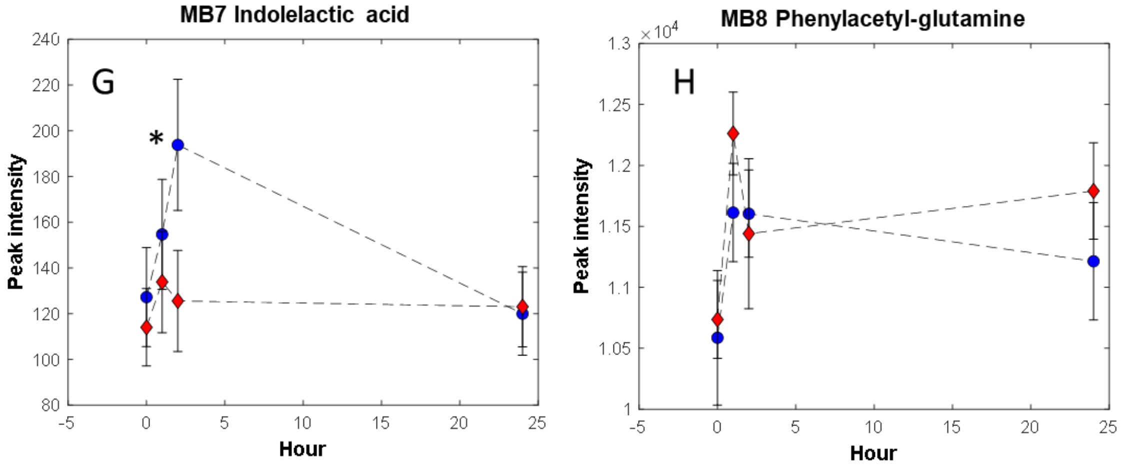

4 Interpretation of the MS/MS spectra

Below is the interpretation of the MS/MS spectra of the tentatively identified markers that were not confirmed with authentic reference standards.

Markers M05 and M06 show a fragmentation pattern that suggests the structure of HydroxyProlyl-Proline and Prolyl-Hydroxyproline, respectively. However, whereas the interpretation is more obvious for M06, M05 requires deeper investigation. The ions at *m/z* 155, 127, and 130 are fragments produced by the breakage of the pyrrolidine rings, whereas the ion 68 is related to the unsaturated pyrrolidine. The fragmentation pattern of the other dipeptides M07, M08, and M09 is also as expected, however none of the dipeptides could be confirmed at level II since they were not found in spectral libraries. Marker M10 Acetyl-Leucine could be identified at level II after matching with mzCloud.

Marker M11 with *m/z* at 246.9944 is a sulfated metabolite of the ion at 167.034, which could fit with several structures such as methoxy-salicylic acid and vanillic acid. However, the MS/MS spectra do not match the spectra reported in mzCloud. M11 does not show any loss of CO_2, whereas the loss of CH_3OH+CO is detected several times and suggests the presence of a methyl ester. This could suggest the structure of dihydroxybenzoic acid methyl ester.

Marker M15 has a fragmentation pattern suggesting 2,3-Dihydro-3,5-dihydroxy-2-oxo-3-indoleacetic acid, but it could not be confirmed at level II since not present in spectral libraries.

M16 at *m/z* 259.0268 suggested the structure of propylparaben sulfate. However, the propylparaben sulfate synthesized by the enzymatic reaction reported in the Material and Methods section did not match either in retention time or in fragmentation with the detected metabolite.

Marker M18 matched with formula $C_6H_8O_3S$. The fragmentation pattern shows the losses of CO_2 and -CH_3 producing the ion at *m/z* 101.004. This ion matches with a structure like dihydrothiophenone (C_4H_6OS). However, the reported phosphite loss is in contrast with this interpretation of the spectrum. No hypothesis could be formulated for the rest of the meat markers which are addressed as unknown.

Marker D01 was associated with Valyl-Proline but assigned to identification level III. Although some detected fragments fit with the simulated spectra (*m/z* 97.005, *m/z* 169.011) or with the spectra available in spectral libraries (*m/z* 100.075, *m/z* 72.981), some other fragments do not. For instance, the ion 116 associated to proline is missing, and the ion *m/z* 128.017 is not reported in the spectral libraries.

Marker D02 is supposed to be a mixture of Prolyl-Leucine, Prolyl-Isoleucine, Leucyl-Proline, Isoleucyl-Proline, due to both the observed chromatographic peak broadening and the fragmentation pattern. By inspecting spectral libraries we can see that *m/z* 183.11 and *m/z* 70.065 belong to the

fragmentation pattern of prolyl-(iso)leucine, and *m/z* 116.070, *m/z* 86.096 and *m/z* 70.065 belong to the fragmentation pattern of (Iso)Leucyl-Proline.

Marker D05 has been tentatively identified as hydroxyphenylacetic acid sulfate with identification confidence level II. Although the MS/MS spectra of the sulfate conjugate are not reported in spectra libraries, the fragments of hydroxyphenyl acetic acid can be found and show the same fragmentation pattern as the identified marker. The confirmation of the retention time was not possible because the sulfate conjugation of this compound was attempted without any results.

Marker D07 has been tentatively identified as Hydroxyphenylacetyl-glutamine. Although 4-hydroxyphenylacetyl-glutamine was chemically synthesized and matched with the fragmentation pattern of the standard, it did not match with the retention time suggesting that the marker is likely the conjugate of another isomer of 4-hydroxyphenylacetic acid.

Marker D10 has been identified at level I because matched with the synthesized standard 4-ethylphenyl sulfate. However, also 3-ethylphenyl sulfate was synthesized and matched with the standard because the chromatographic method employed in this work was not able to separate the two isomers. 4-ethylphenyl sulfate was the final assigned identity since the chromatographic shape of the peak was more similar, but 3-ethylphenyl sulfate cannot be totally excluded. 2-ethylphenyl sulfate did not match.

Marker D11 has been tentatively identified as acetamido octanoic acid, commonly known as acetamidocaprylic acid. Although all the detected fragments have been explained, no experimental spectra are available in the literature, therefore the marker has been associated to identification confidence level III.

Another class of tentatively identified metabolites is a series of pyrrolidine-based structures, i.e. D12, D13, D14 and D15. A similar fragmentation pattern was detected for the metabolites, where D14 and D15 are two conjugates of the main metabolites D12 and D13 with a mass difference of 99.0701, that was associated to the structure of piperidone (C_5H_9NO). The fragmentation pattern of the two isomers D12 and D13 cannot be distinguished due to the overlapping of the chromatographic peaks. Several fragments, such as $[C_8H_9]^+$ $[C_8H_7]^+$ $[C_7H_7]^+$ $[C_6H_7]^+$, suggest the presence of an aromatic ring. Based on a previous analysis of the fragmentation spectra the compounds D12 and D13 were tentatively identified as Propyl-Hydroxy-Tetrahydroquinoline carboxylic acid and its isomer. This compound is similar to kynurenic acid, that is a product of the metabolism of tryptophan. Considering that tryptophan has been identified as a biomarker of dairy products, this hypothesis looked very plausible. However, the highly abundant fragment *m/z* 119.073, deriving from the loss of *m/z* 70.065 that is typical of pyrrolidines, suggested that the most likely structure was Pyrrolidine-ethyl-hydroxy benzoic acid. Based on the fragmentation pattern the ethyl group must be linked to the benzoic acid aromatic ring. The hypothesis of a bond between the carboxylate and the pyrrolidine, representing the structure of proline, was considered, but the proline fragment was not detected.

Marker D17 has been tentatively identified at level III as ethyl-methylphenyl sulfate, even though the structure of prolyl-phenol sulfate cannot be excluded.

Marker D19 has been tentatively identified as a conjugate of hydroxy-indoleacetic acid, however, since the fragmentation pattern could not be entirely verified, it has been classified as an unknown biomarker.

Marker D23 was tentatively identified at level III as angelyl-glycine since it shows the glycine fragment but was not found in spectral libraries.

Marker MB4, has been tentatively identified as indoxyl sulfate. Since it does not show informative fragments, it has been assigned with level III identification.
